# Host–Guest Inversion Engineering Induced Superionic Composite Solid Electrolytes for High-Rate Solid-State Alkali Metal Batteries

**DOI:** 10.1007/s40820-025-01691-7

**Published:** 2025-03-17

**Authors:** Xiong Xiong Liu, Long Pan, Haotian Zhang, Pengcheng Yuan, Mufan Cao, Yaping Wang, Zeyuan Xu, Min Gao, Zheng Ming Sun

**Affiliations:** School of Materials Science and Engineering, Southeast University, Nanjing, 211189 People’s Republic of China

**Keywords:** Host–guest inversion engineering, SiO_2_ nanoparticle, Superionic conductivity, Composite solid electrolyte, Solid-state alkali metal battery

## Abstract

Composite solid electrolytes (CSEs) are promising for solid-state Li metal batteries but suffer from inferior room-temperature ionic conductivity due to sluggish ion transport and high cost due to expensive active ceramic fillers. Here, a host–guest inversion engineering strategy is proposed to develop superionic CSEs using cost-effective SiO_2_ nanoparticles as passive ceramic hosts and poly(vinylidene fluoride-hexafluoropropylene) (PVH) microspheres as polymer guests, forming an unprecedented “polymer guest-in-ceramic host” (i.e., PVH-in-SiO_2_) architecture differing from the traditional “ceramic guest-in-polymer host”. The PVH-in-SiO_2_ exhibits excellent Li-salt dissociation, achieving high-concentration free Li^+^. Owing to the low diffusion energy barriers and high diffusion coefficient, the free Li^+^ is thermodynamically and kinetically favorable to migrate to and transport at the SiO_2_/PVH interfaces. Consequently, the PVH-in-SiO_2_ delivers an exceptional ionic conductivity of 1.32 × 10^−3^ S cm^−1^ at 25 °C (vs*.* typically 10^−5^–10^−4^ S cm^−1^ using high-cost active ceramics), achieved under an ultralow residual solvent content of 2.9 wt% (vs*.* 8–15 wt% in other CSEs). Additionally, PVH-in-SiO_2_ is electrochemically stable with Li anode and various cathodes. Therefore, the PVH-in-SiO_2_ demonstrates excellent high-rate cyclability in LiFePO_4_|Li full cells (92.9% capacity-retention at 3C after 300 cycles under 25 °C) and outstanding stability with high-mass-loading LiFePO_4_ (9.2 mg cm^−1^) and high-voltage NCM622 (147.1 mAh g^−1^). Furthermore, we verify the versatility of the host–guest inversion engineering strategy by fabricating Na-ion and K-ion-based PVH-in-SiO_2_ CSEs with similarly excellent promotions in ionic conductivity. Our strategy offers a simple, low-cost approach to fabricating superionic CSEs for large-scale application of solid-state Li metal batteries and beyond.

**Supplementary Information:**

The online version contains supplementary material available at 10.1007/s40820-025-01691-7.

## Highlights

- Host–guest inversion engineering is proposed to create poly(vinylidene fluoride-hexafluoropropylene) (PVH)-in-SiO_2_ composite solid electrolytes with an original “polymer guest-in-ceramic host” architecture, exhibiting optimized interfacial contacts and comprehensive properties.
- The PVH-in-SiO_2_ exhibits an overwhelming ionic conductivity of 1.32 × 10^−3^ S cm^−1^ at 25 °C, with an ultralow residual solvent content of 2.9 wt%. In addition, the LiFePO_4_|PVH-in-SiO_2_|Li full cells deliver a significant capacity retention of 92.9% at an ultrahigh rate of 3C after 300 cycles at 25 °C.
- The host–guest inversion engineering is a versatile strategy, as proved by preparing Na^+^ and K^+^-based PVH-in-SiO_2_ composite solid electrolytes, delivering excellent ionic conductivity of 10^−4^ S cm^−1^ at 25 °C (vs. 10^−6^–10^−5^ S cm^−1^ of previous reports).

**Supplementary Information:**

The online version contains supplementary material available at 10.1007/s40820-025-01691-7.

## Graphical abstract

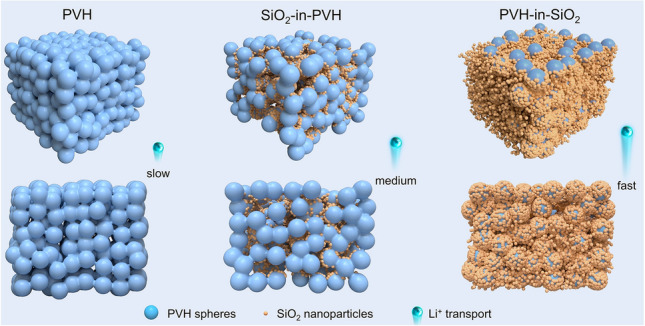

## Introduction

The ever-growing anxieties about the battery life and safety of liquid lithium-ion batteries are boosting the flourishing development of solid-state Li metal batteries as they can simultaneously improve energy density and inhibit thermal runaway [1–3]. Solid-state electrolytes are fundamental to solid-state Li metal batteries, requiring decent interfacial contacts, high mechanical modulus, wide electrochemical window, and, in particular, high ionic conductivity (at least 10^−3^ S cm^−1^ at room temperature) to meet practical applications [4–6]. In this sense, composite solid-state electrolytes (CSEs), which are commonly comprised of active ceramics as fillers (i.e., guests) and polymers as matrices (i.e., hosts) to form traditional “ceramic guest-in-polymer host” architectures, have provoked significant interest. Because active ceramic guests are ion-conducting, CSEs can integrate the high modulus and ionic conductivity of active ceramic guests with the excellent processability and interfacial compatibility of polymer hosts [7, 8]. To date, various active ceramic guests have been introduced, including oxides (e.g., Li_7_La_3_Zr_2_O_12_) [9–13], sulfides (e.g., Li_6_PS_5_Cl) [14, 15], and phosphates (e.g., Li_1.3_Al_0.3_Ti_1.7_(PO_4_)_3_) [16, 17]. However, CSEs based on active ceramic guests are still far from practical applications because of their insufficient ionic conductivities (generally 10^−5^–10^−4^ S cm^−1^ at room temperature) [18–20]. Another critical challenge is induced by the high cost, harsh synthesis, and complex handling of active ceramic guests [21, 22].

To address these problems, passive ceramics, which are not ion conductors, have been proposed as guests to incorporate with polymer hosts, including SiO_2_, Al_2_O_3_, TiO_2_, etc.[23, 24]. Compared to active ceramics, passive ceramics possess advantages such as lower cost, simpler preparation, easier handling, and better environmental compatibility. These merits make passive ceramics particularly appealing for large-scale applications of CSEs [25]. More importantly, CSEs based on passive ceramics exhibit comparable ionic conductivities (10^−5^–10^−4^ S cm^−1^ at room temperature) to those based on active ceramics [26, 27]. Therefore, it is of significant importance to develop next-generation passive ceramic-based CSEs to achieve superionic conductivity (at least 10^−3^ S cm^−1^) at room temperature.

Although passive ceramics are not ion-conducting, they have strong interfacial Lewis acid–base interactions with Li salts, thereby expediting the dissociation of Li salts [28]. They can also weaken polymer crystallization, producing more amorphous regions to boost Li^+^ transport [29]. Furthermore, recent theoretical calculations revealed that the interfacial ionic conductivity between ceramics and polymers can reach as high as 10^−2^ S cm^−1^ (at 30 °C) [30]. These findings, therefore, inspire that maximizing the ceramic/polymer interfacial contacts can promise adequate moveable ions with superionic conductivities at room temperature. In this circumstance, increasing the weight ratio of ceramic guests and polymer hosts (up to 400 wt%) has been proposed to increase the interfacial contacts [31]. Despite the high content of ceramic guests, only conventional “ceramic guest-in-polymer host” architectures were obtained with limited interfacial contacts (Fig. S1), because ceramics typically have much higher densities than polymers and exhibit micron-order particle sizes (Table S1). Accordingly, increasing the content of ceramic guests only leads to limited progress in ionic conductivity (10^−4^ S cm^−1^ at room temperature) [31–36]. Therefore, building advanced CSEs with novel host–guest architectures to achieve optimized interfacial contacts and continuous ion pathways is a fundamental challenge rather than simply improving the content of passive ceramic guests.

**Fig. 1:**
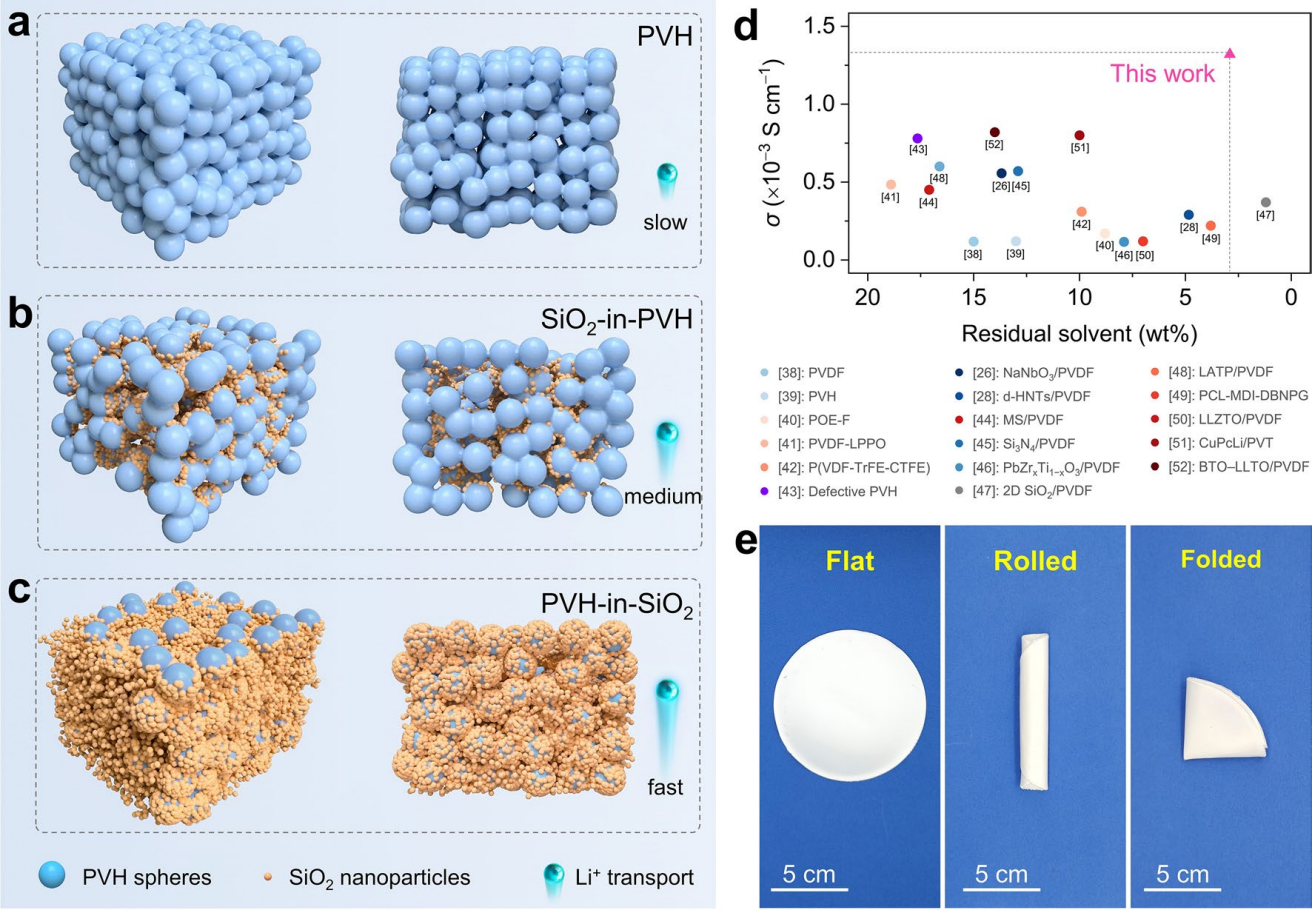
Structure and ion transport performance of PVH-in-SiO_2_. **a**–**c** Illustration of structures and ion-conducting behaviors of **a** PVH, **b** SiO_2_-in-PVH, and **c** PVH-in-SiO_2_. The PVH forms interconnected micron-sized spheres (blue balls), in which SiO_2_ nanoparticles (orange balls) are filled into the voids, forming SiO_2_-in-PVH or PVH-in-SiO_2_ architectures by varying the SiO_2_ content. Note that ions transport sluggishly inside the polymer but show high theoretical conductivity (up to 10^−2^ S cm^−1^) at the ceramic/polymer interfaces [30]. Therefore, the PVH-in-SiO_2_ shows fast ion diffusion at the SiO_2_/PVH interfaces because PVH spheres are densely covered by SiO_2_ nanoparticles, forming continuous interfacial ion-conducting highways. **d** Comparison of ionic conductivity (at 25 °C) and residual solvent content of our PVH-in-SiO_2_ with other reported CSEs and PSEs [26, 28, 38–52]. Although the residual solvent is favorable for ion conduction, it seriously affects Li metal anodes and battery safety. Therefore, our PVH-in-SiO_2_ efficiently addresses the residual solvent problems without compromising the superionic capability. **e** Digital photographs of a sizeable and flexible PVH-in-SiO_2_ film with a diameter of 11 cm. (Color figure online)

In this contribution, we, for the first time, propose a host–guest inversion engineering to realize superionic CSEs composed of monodisperse SiO_2_ nanoparticles and poly(vinylidene fluoride-hexafluoropropylene) (PVH) microspheres. Note that SiO_2_ is a typical passive ceramic and serves as the host in our case rather than the traditional guest because of its relatively low density to boost the volume-to-weight ratio (Table S1). In addition, SiO_2_ shows the merits of low cost, facile preparation, and environmental friendliness. Correspondingly, PVH serves as the guest instead of the traditional host because it favors forming interconnected sphere scaffolds (Fig. 1a). In particular, PVH exhibits a relatively high density that can lower its volume-to-weight ratio, leaving continuous voids with large volumes for filling SiO_2_ nanoparticle hosts. Moreover, PVH shows high mechanical strength and excellent dielectric constant that facilitates the dissociation of alkali metal salt [37]. As a result, the obtained CSEs undergo an architecture inversion from the traditional “ceramic guest-in-polymer host” (i.e., “SiO_2_-in-PVH”) (Fig. 1b) to the innovative “polymer guest-in-ceramic host” (i.e., “PVH-in-SiO_2_”) (Fig. 1c) when the SiO_2_/PVH weight ratio increases from 20/100 to 70/100. Such ratio is much lower than previous reports, which have a ceramic/polymer weight ratio of up to 400/100. For simplification and clarification, the terms “PVH”, “SiO_2_-in-PVH”, and “PVH-in-SiO_2_” are used throughout the manuscript for the CSEs with SiO_2_/PVH weight ratio of 0/100, 20/100, and 70/100, respectively.

Benefiting from the unprecedented PVH-in-SiO_2_ architecture, the PVH microspheres are densely covered by numerous SiO_2_ nanoparticles, maximizing the SiO_2_/PVH interfacial contacts and creating continuous interfacial pathways for fast ion transport (Fig. 1c). As a result, the PVH-in-SiO_2_ delivers an unexceptionally high ionic conductivity of 1.32 × 10^−3^ S cm^−1^ at 25 °C under an extremely low residual solvent content (2.9 wt%, calculated based on the total weight loss from ambient temperature to 200 °C), overwhelming most recently reported CSEs and polymer solid-state electrolytes (PSEs) (Fig. 1d and Table S2) [26, 28, 38–52]. The PVH-in-SiO_2_ also features easy handling, flexibility, and scalability (Fig. 1e), which is essential for their large-scale implementation in practical solid-state Li metal batteries. In addition, the PVH-in-SiO_2_ shows outstanding electrochemical stability against Li metal with a cycling life of more than 1000 h at the current density of 0.2 mA cm^−2^. Moreover, the LiFePO_4_|PVH-in-SiO_2_|Li solid-state full cells exhibit excellent rate capability and cycling stability, with a remarkable capacity retention of 92.9% at a high current density of 3C after 300 cycles under 25 °C, surpassing many recently reported LiFePO_4_|Li solid-state full cells. The PVH-in-SiO_2_ also demonstrates good cyclability and safety when using high-mass-loading LiFePO_4_ (9.2 mg cm^−2^) and high-voltage NCM622 cathodes (147.1 mAh g^−2^ at 0.2C after 100 cycles). More impressively, we verify that the host–guest inversion engineering strategy is general and can be extended to preparing advanced Na-ion and K-ion-based CSEs by simply replacing Li salt with its counterparts (i.e., Na and K salts). The resulting PVH-in-SiO_2_-Na and PVH-in-SiO_2_–K exhibit exciting ionic conductivities of 3.03 × 10^−4^ and 2.56 × 10^−4^ S cm^−1^ (14 and 64 times that of PVH-Na and PVH-K at 25 °C), respectively. The PVH-in-SiO_2_-Na and PVH-in-SiO_2_**-**K also showcase outstanding full-cell cycling stability with 86.4% and 84.3% capacity retention after 500 cycles at the current density of 0.5C under 25 °C. Our host–guest inversion engineering strategy opens a new avenue for using passive ceramics to develop low-cost and scalable high-performance CSEs, promising the practical applications of next-generation solid-state alkali metal batteries.

## Results and Discussion

### Synthesis, Morphology, and Properties of PVH-in-SiO_2_

**Fig. 2:**
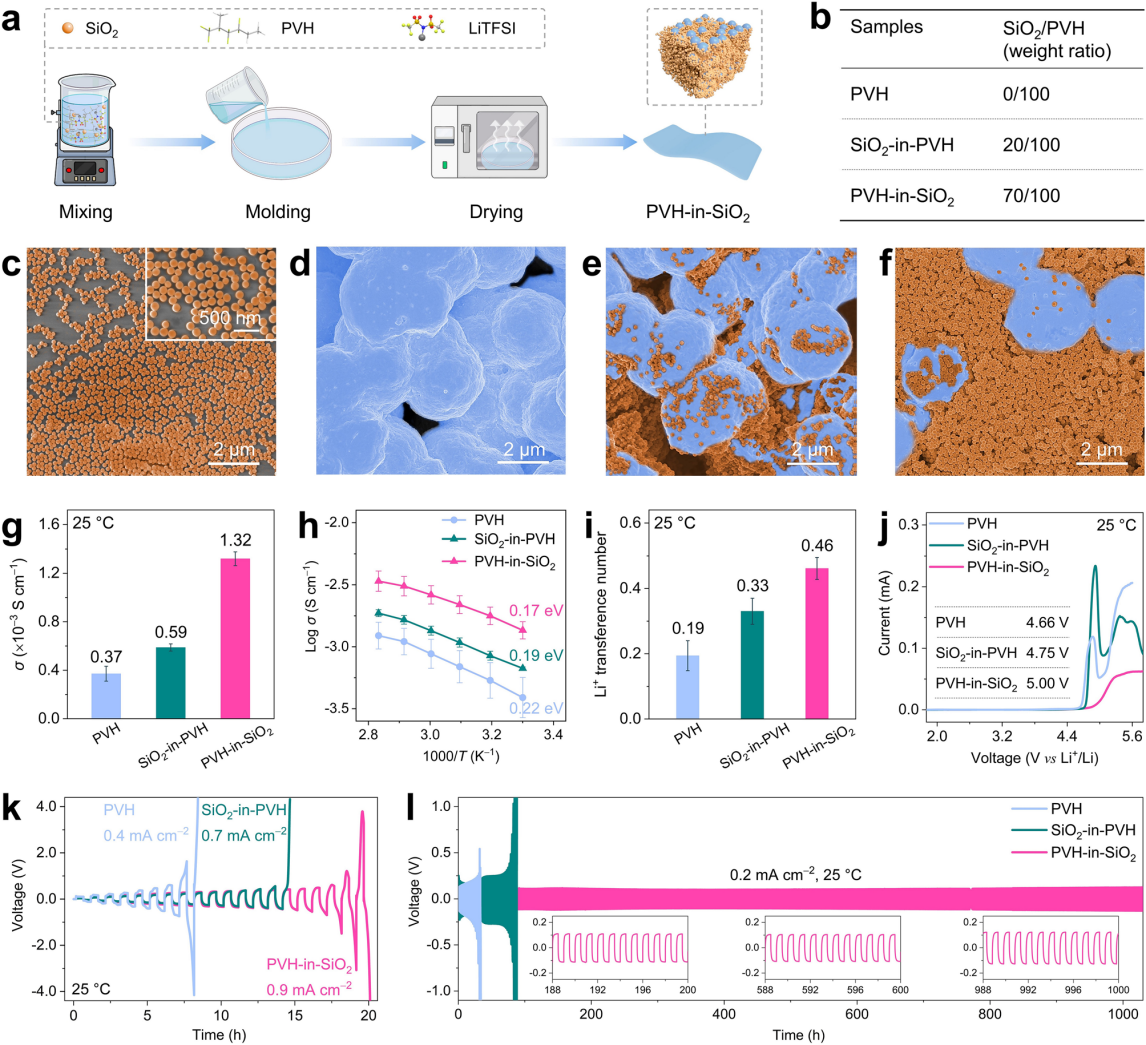
Morphology, ion conduction, and electrochemical stability of PVH-in-SiO_2_. **a** Schematic of the preparation process of PVH-in-SiO_2_. The term LiTFSI represents lithium bis(trifluoromethanesulfonyl)imide. **b** SiO_2_/PVH weight ratios for PVH, SiO_2_-in-PVH, and PVH-in-SiO_2_. Note that the LiTFSI/PVH ratios of all three samples were kept at 100/100 wt%. **c**, SEM images of SiO_2_ nanoparticles. **d–f** Top-view SEM images of **d** PVH, **e** SiO_2_-in-PVH, and **f** PVH-in-SiO_2_ CSEs. The blue and orange represent the PVH spheres and SiO_2_ nanoparticles, respectively. **g–i** Li^+^ conducting properties: **g** ionic conductivities (*σ*), **h** Arrhenius plots between *σ* and temperature (*T*), **i** Li^+^ transference numbers of PVH, SiO_2_-in-PVH, and PVH-in-SiO_2_ CSEs. **j**–**l** Electrochemical stability against Li metal anodes: **j** LSV curves, **k** CCD values (Li plating/stripping time: 0.5 h per step; current density increment: 0.05 mA cm^−2^), and **l** galvanostatic voltage profiles of Li||Li symmetric cells at 0.2 mA cm^−2^ (1 h per step) of PVH, SiO_2_-in-PVH, and PVH-in-SiO_2_. All measurements were carried out at 25 °C, except that the Arrhenius plots were collected from 30 to 80 °C

The preparation process is depicted in Fig. 2a, b, before which monodisperse SiO_2_ nanoparticles with an average diameter of 158 nm were synthesized by an efficient hydrolysis approach (Figs. 2c and S2, S3) [53]. PVH-in-SiO_2_ films were fabricated using a simple solution casting approach, through which the SiO_2_ nanoparticles and PVH were mixed at the weight ratio of 70/100. Control samples, viz., PVH and SiO_2_-in-PVH films, were also prepared using the same procedure, except that the SiO_2_/PVH weight ratios were 0/100 and 20/100, respectively.

To reveal the morphological information of the as-prepared CSE films, scanning electron microscopy (SEM) was employed. Figures 2d and S4, S5 show the SEM images of PVH, in which 2.9 µm spheres are formed and interconnected, leaving abundant voids. When a small ratio of SiO_2_ nanoparticles is introduced (i.e., SiO_2_/PVH = 20/100), they partially occupy the voids and loosely cover the PVH spheres (Figs. 2e and S6, S7). As a result, a traditional “creamic guest-in-polymer host” architecture (i.e., SiO_2_-in-PVH in our case) is generated, leading to insufficient and discontinuous PVH/SiO_2_ interfacial contacts that are unfavorable for fast Li^+^ conducting (as discussed later). As the SiO_2_/PVH weight ratio increases to 70/100, the PVH spheres are immersed in the “sea” of SiO_2_ nanoparticles, forming an innovative “polymer host-in-creamic guest” architecture (i.e., PVH-in-SiO_2_ in our case), as Figs. 2f and S8, S9 show. With this architecture, the PVH surfaces are entirely covered by SiO_2_ nanoparticles, resulting in adequate and continuous interfacial contacts and pathways for fast Li^+^ conduction.

The superionic properties of PVH-in-SiO_2_ were comprehensively evaluated by investigating its ionic conductivity, activation energy, and Li^+^ transference number. Figures 2g and S10 show that the PVH-in-SiO_2_ exhibits an outstanding ionic conductivity of 1.32 × 10^−3^ S cm^−1^ at 25 °C, which is an order of magnitude higher than PVH (3.7 × 10^−4^ S cm^−1^) and SiO_2_-in-PVH (5.9 × 10^−4^ S cm^−1^). The ionic conductivity results indicate that the PVH-in-SiO_2_ architecture can significantly facilitate the LiTFSI dissociation and ion mobility, which is further validated by calculating activation energies using the Arrhenius equation (Figs. 2h and S11). The PVH has a high activation energy of 0.22 eV, which quickly declined to 0.17 eV in the case of PVH-in-SiO_2_, implying that PVH-in-SiO_2_ possesses the lowest energy barrier for ion mobility. This energy barrier is also smaller than those reported previously (Table S3) [26, 28, 38–43, 45–48, 50, 52]. In addition, the PVH-in-SiO_2_ also has a large Li^+^ transference number of 0.46, which is approximately 2.4 and 1.4 times that of PVH and SiO_2_-in-PVH (Figs. 2i and S12), suggesting that the PVH-in-SiO_2_ architecture is more favorable for boosting the mobility of Li^+^ instead of its counterion (i.e., TFSI^−^) [54]. Moreover, we stress that the superionic properties of PVH-in-SiO_2_ are not attributed to the residual solvent because the total residual solvent is only 2.9 wt% (Fig. S13), which is significantly lower than previous reports (generally > 10 wt%, Fig. 1d and Table S2) [26, 28, 38–52]. Such a low residual solvent content can minimize the side reactions between solvent and Li metal anodes, thereby achieving good cycling stability in both Li-based symmetric and full cells (as proved and discussed later). In other words, our PVH-in-SiO_2_ efficiently addresses the residual solvent problems without compromising the fast-ion-conducting capability.

The electrochemical oxidation stability of PVH-in-SiO_2_ was initially investigated using linear sweeping voltammetry (LSV) at 25 °C, and the results are shown in Fig. 2j. In the case of PVH, a negligible oxidation peak is observed at approximately 4.00 V, after which the current starts to roar at 4.66 V, indicating that fast oxidation occurs. After introducing SiO_2_ nanoparticles, the small oxidation peak disappears, and the fast oxidation voltage rises to 4.75 and 5.00 V in the cases of SiO_2_-in-PVH and PVH-in-SiO_2_, respectively. These findings imply that the PVH-in-SiO_2_ exhibits robust electrochemical oxidation stability (Table S3) [26, 28, 38–43, 45–48, 50, 52], showing good potential for pairing with high-voltage cathodes to realize high-energy–density solid-state Li metal batteries. We also evaluated the electrochemical stability of PVH-in-SiO_2_ against Li metal anode by assembling Li|Li symmetric cells. Figure 2k shows the critical current density (CCD) curves, in which the PVH-in-SiO_2_ exhibits a high CCD of 0.90 mA cm^−2^. In contrast, the PVH and SiO_2_-in-PVH only show a low CCD of 0.40 and 0.70 mA cm^−2^, respectively. In addition, their long-term electrochemical stability against Li metal anode is also investigated at different current densities (Figs. 2l and S14). The PVH-in-SiO_2_ remains stable for over 1000 h at the current densities of 0.1 and 0.2 mA cm^−2^, with corresponding overpotentials of only 68.2 and 88.6 mV, respectively. On the contrary, the SiO_2_-in-PVH quickly fails after 330 and 88 h at 0.1 and 0.2 mA cm^−2^, respectively. Similar poor stability is also observed in the case of PVH (51 and 33 h at 0.1 and 0.2 mA cm^−2^, respectively). The poor stability of PVH not only originates from the sluggish ion conduction but is also attributed to its chemical instability against Li metal anodes [44]. Quantitatively, the total Li stripping/plating capacity of PVH-in-SiO_2_-based symmetric cells is over 200 mAh cm^−2^, which is more competitive than those using other CSEs and PSEs (Table S3) [26, 28, 38–43, 45–48, 50, 52].

Other parameters that influence the properties of PVH-in-SiO_2_ are also examined. For instance, if the SiO_2_/PVH weight ratio is further increased (e.g., 100/100), it is hard to form mechanically durable CSE films (Fig. S15); if the size of SiO_2_ nanoparticles is increased (e.g., 488 nm), the sphere morphology of PVH disappears, and ionic conductivity declines to 1.2 × 10^−4^ S cm^−1^ at 25 °C (Figs. S16 and S17).

To summarize, we have successfully realized an innovative PVH-in-SiO_2_ (i.e., “polymer guest-in-ceramic host”) architecture with superionic conductivity (1.32 × 10^−3^ S cm^−1^ at 25 °C), low activation energy (0.17 eV), and electrochemical stability by introducing 158 nm SiO_2_ nanoparticles into the interconnected PVH spheres with a SiO_2_/PVH weight ratio of 70/100.

### Li^+^ Transport Mechanism of PVH-in-SiO_2_

**Fig. 3:**
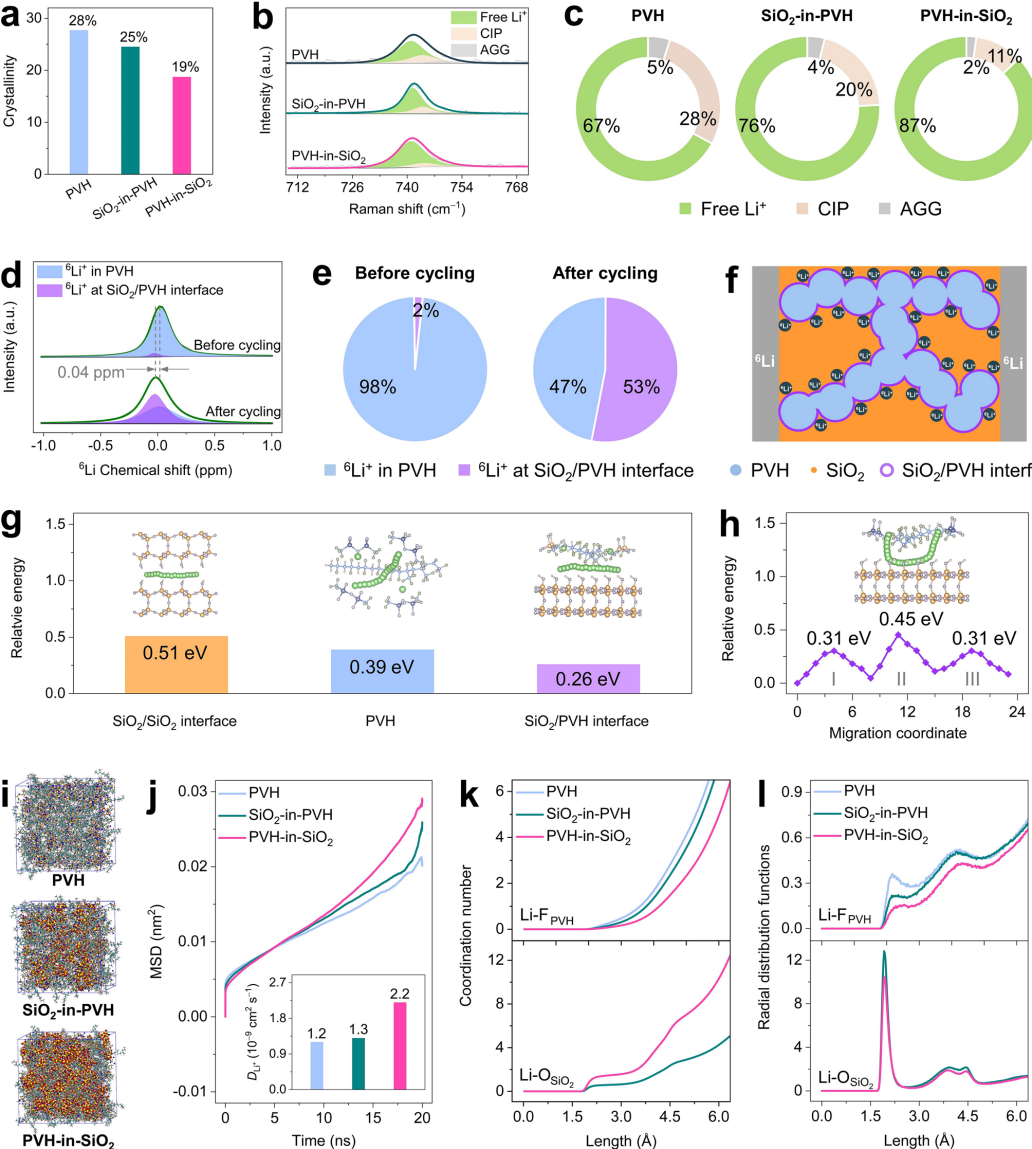
Li^+^ transport mechanism of PVH-in-SiO_2_. **a**–**c** Structural and spectral analyses of PVH, SiO_2_-in-PVH, and PVH-in-SiO_2_: **a** crystallinity, **b** Raman spectra, and **c** ratios of free TFSI^−^, CIP, and AGG. **d** ^6^Li SSNMR spectra of PVH-in-SiO_2_ before and after plating/stripping. **e** Quantification results of ^6^Li^+^ amount in the PVH and at the SiO_2_/PVH interfaces of PVH-in-SiO_2_ before and after plating/stripping. **f** Schematic of ^6^Li^+^ migration and transport in PVH-in-SiO_2_. **g** Calculation models (insets) and energy barriers of Li^+^ transport along the SiO_2_/SiO_2_ interfaces, PVH chains, and SiO_2_/PVH interfaces. **h** Calculation models (inset) and energy barriers of dynamic Li^+^ migration process from PVH to SiO_2_ and back to PVH (Stage I, II, and III). **i–l** MD simulation results of PVH, SiO_2_-in-PVH, and PVH-in-SiO_2_: **i** Model snapshots, **j** MSD curves, **k** coordination numbers, and **l** radial distribution functions

We initially employed X-ray diffraction (XRD) to determine the crystallinity, and the XRD patterns are shown in Fig. S18. Owing to the highly disordered structure of the SiO_2_ nanoparticles, the two primary reflections at 20.2° and 38.8° can be assigned to the crystalline domain of PVH [39]. As the SiO_2_ nanoparticle content rises, the reflection intensity of these two peaks gradually lessens. Consequently, the PVH-in-SiO_2_ exhibits a much lower crystallinity of 19% than PVH (28%) and SiO_2_-in-PVH (25%), as Fig. 3a discloses. Therefore, the PVH-in-SiO_2_ has more amorphous regions than PVH and SiO_2_-in-PVH, which are advantageous to fast Li^+^ transport [55].

We subsequently utilized Raman spectroscopy to elucidate the dissociation of LiTFSI (Fig. 3b). Three peaks at 741, 744, and 749 cm^−1^ can be distinguished, corresponding to the free TFSI^−^, contact ion pairs (CIP), and aggregated ion pairs (AGG), respectively [56]. Among them, the free TFSI^−^ anions are formed from the dissociation of LiTFSI, while the CIP and AGG represent the TFSI^−^ anions associated with one or more Li^+^ cations, respectively [39]. In other words, the higher the free TFSI^−^ content is, the higher the dissociation degree of LiTFSI is. Quantitatively, the PVH-in-SiO_2_ has the highest free TFSI^−^ content of 87%, which is 1.3 and 1.1 times that of PVH (67%) and SiO_2_-in-PVH (76%), as Fig. 3c depicts. These results suggest that the PVH-in-SiO_2_ architecture can significantly facilitate the dissociation of LiTFSI, providing more moveable Li^+^ for enhancing ionic conductivity [57]. This conclusion is further proved by calculating the dissociation energy of LiTFSI on SiO_2_ surface, PVH chain, and SiO_2_/PVH interface via density functional theory (DFT) (Fig. S19). The SiO_2_/PVH interface shows the lowest dissociation energy of 0.72 eV compared to SiO_2_ surface (2.46 eV) and PVH chain (1.36 eV), indicating that the SiO_2_/PVH interface can effectively promote LiTFSI dissociation.

To reveal the Li^+^ transport channel in PVH-in-SiO_2_, we performed solid-state nuclear magnetic resonance (SSNMR) spectroscopy by assembling ^6^Li symmetric cells using PVH-in-SiO_2_ as CSEs. The ^6^Li symmetric cells were cycled at a stripping/plating current of 50 µA for 10 cycles (Fig. S20), upon which a portion of ^7^Li^+^ in the PVH-in-SiO_2_ was replaced by ^6^Li^+^. We observe that the pristine PVH-in-SiO_2_ exhibits a ^6^Li resonance at *ca*. 0.02 ppm, which shifts to *ca*. − 0.02 ppm after cycling tests (Fig. 3d), indicating the change of ^6^Li^+^ chemical environments [58]. More specifically, the resonance peak can be further fitted into two peaks at approximately 0.02 ppm and − 0.03 ppm. The former peak (0.02 ppm) belongs to the ^6^Li^+^ in the PVH, while the latter one (− 0.03 ppm) represents the ^6^Li^+^ at the SiO_2_/PVH interfaces (see details in Fig. S21). Quantitatively, the ^6^Li^+^ ratio in the PVH shows a tremendous decline from 98% to 47% after cycling, corresponding to a dramatical increase of the ^6^Li^+^ ratio at the SiO_2_/PVH interfaces from 2% to 53% (Fig. 3e). These SSNMR results suggest that the Li^+^ in the PVH-in-SiO_2_ has a strong tendency to migrate from the PVH to the SiO_2_/PVH interfaces and subsequently transports at the SiO_2_/PVH interfaces upon in-service operation, as Fig. 3f illustrates.

We conducted DFT calculations to elucidate why Li^+^ tends to migrate to and transport at the SiO_2_/PVH interfaces in the case of PVH-in-SiO_2_. Generally, there are three routes for Li^+^ transport, including SiO_2_/SiO_2_ interfaces, PVH chains, and SiO_2_/PVH interfaces (insets of Fig. 3g). On the one hand, the Li^+^ transport at the SiO_2_/SiO_2_ interfaces has the highest energy barrier of 0.51 eV (Fig. 3g), suggesting that the SiO_2_/SiO_2_ interfaces are thermodynamically unfavorable for Li^+^ transport. This finding is in good agreement with the SSNMR results that no ^6^Li^+^ peak at the SiO_2_/SiO_2_ interfaces is found. On the other hand, the energy barriers of Li^+^ transport along the PVH chain and at the SiO_2_/PVH interfaces are significantly reduced to 0.39 and 0.26 eV, respectively, indicating that SiO_2_/PVH interfaces are the most energetic route for Li^+^ transport. We further model the dynamic Li^+^ migration process from PVH to SiO_2_ and then back to PVH. In detail, this process involves three steps (inset of Fig. 3h): Li^+^ migration from the PVH chain to the SiO_2_ surface (Stage I), Li^+^ transport along the SiO_2_ surface (Stage II), and Li^+^ migration from the SiO_2_ surface to the PVH chain (Stage III). Figure 3h showcases that the energy barriers of Step I and Step III (0.31 eV) are much lower than that of Step II (0.45 eV). In addition, they are also lower than that of Li^+^ transport along the PVH chain (0.39 eV, Fig. 3g). These results demonstrate that Li^+^ in the PVH thermodynamically tends to migrate to the SiO_2_/PVH interfaces and transport along the continuous interfacial highways constructed by the PVH-in-SiO_2_ architecture.

In addition to the atomic scale DFT calculations, we further utilized molecular dynamics (MD) simulations to disclose the Li^+^ transport behavior in PVH-in-SiO_2_ on a larger scale. Figure 3i presents the snapshots of MD simulation models of PVH, SiO_2_-in-PVH, and PVH-in-SiO_2_, which have volumes of 207, 225, and 278 nm^3^, respectively. The mean squared displacements (MSD) curves of these three samples were simulated and displayed in Fig. 3j. The MSD curve becomes steeper as more SiO_2_ nanoparticles are introduced. Quantitatively, we can calculate the corresponding Li^+^ diffusion coefficient (${\text{D}}_{{\text{Li}}^{+}}$) from MSD curves because it is proportional to the curve slope. (Time range between 10 and 18 ns is used.) The PVH exhibits a low ${\text{D}}_{{\text{Li}}^{+}}$ of 1.2 × 10^−9^ cm^2^ s^−1^, which nearly doubles in the case of PVH-in-SiO_2_ (2.2 × 10^−9^ cm^2^ s^−1^, inset of Fig. 3j), demonstrating that the PVH-in-SiO_2_ has a much better Li^+^ transport kinetics than PVH and SiO_2_-in-PVH. In addition, the coordination environment of Li^+^ was analyzed to demonstrate its transport at the SiO_2_/PVH interfaces. Figure 3k shows the coordination numbers between Li^+^ and F atoms of PVH (labeled as Li-F_PVH_) and between Li^+^ and O atoms of SiO_2_ (labeled as ${\text{Li-O}}_{{\text{SiO}}_{2}}$). As the SiO_2_ content increases, the coordination numbers of Li-F_PVH_ decreases, while the coordination numbers of ${\text{Li-O}}_{{\text{SiO}}_{2}}$ increases. These results suggest that Li^+^ is favorable to migrating to the SiO_2_/PVH interfaces, which is further validated by the radial distribution functions (Fig. 3l). For instance, the dominant peak of Li-F_PVH_ of PVH-in-SiO_2_ locates at 2.54 Å, which is higher than those of PVH (2.18 Å) and SiO_2_-in-PVH (2.36 Å). The peak shift to longer distances can be attributed to the Li^+^ migration to the SiO_2_/PVH interfaces.

Finally, we conduct the finite element analysis to simulate ion transport in a PVH-in-SiO_2_ model with a simulation calculation area of 18 µm × 18 µm (Fig. S22a), further revealing the impact of SiO2/PVH interfaces on Li^+^ transport. Figure S22b is the distribution of current densities in the PVH-in-SiO_2_ model. The SiO_2_/PVH interface exhibits a high current density of 10^2^–10^3^ mA cm^−2^, whereas the current density in PVH is less than 10^2^ mA cm^−2^. This result suggests that the SiO_2_/PVH interface provides highways for ion transport and plays a dominant role in the ion transport process.

To summarize, the PVH-in-SiO_2_ architecture significantly decreases the crystallinity of the PVH guest, providing sufficient amorphous areas for more accessible Li^+^ transport in the PVH. In addition, the PVH-in-SiO_2_ architecture also facilitates the dissociation of LiTFSI, achieving high-concentration free Li^+^. These Li^+^ cations are thermodynamically and kinetically favorable to migrate to and transport at the continuous SiO_2_/PVH interfaces because of their low energy barriers and high diffusion coefficient, resulting in the superionic properties of PVH-in-SiO_2_.

### Performance of Solid-State Full Cells Based on PVH-in-SiO_2_

**Fig. 4:**
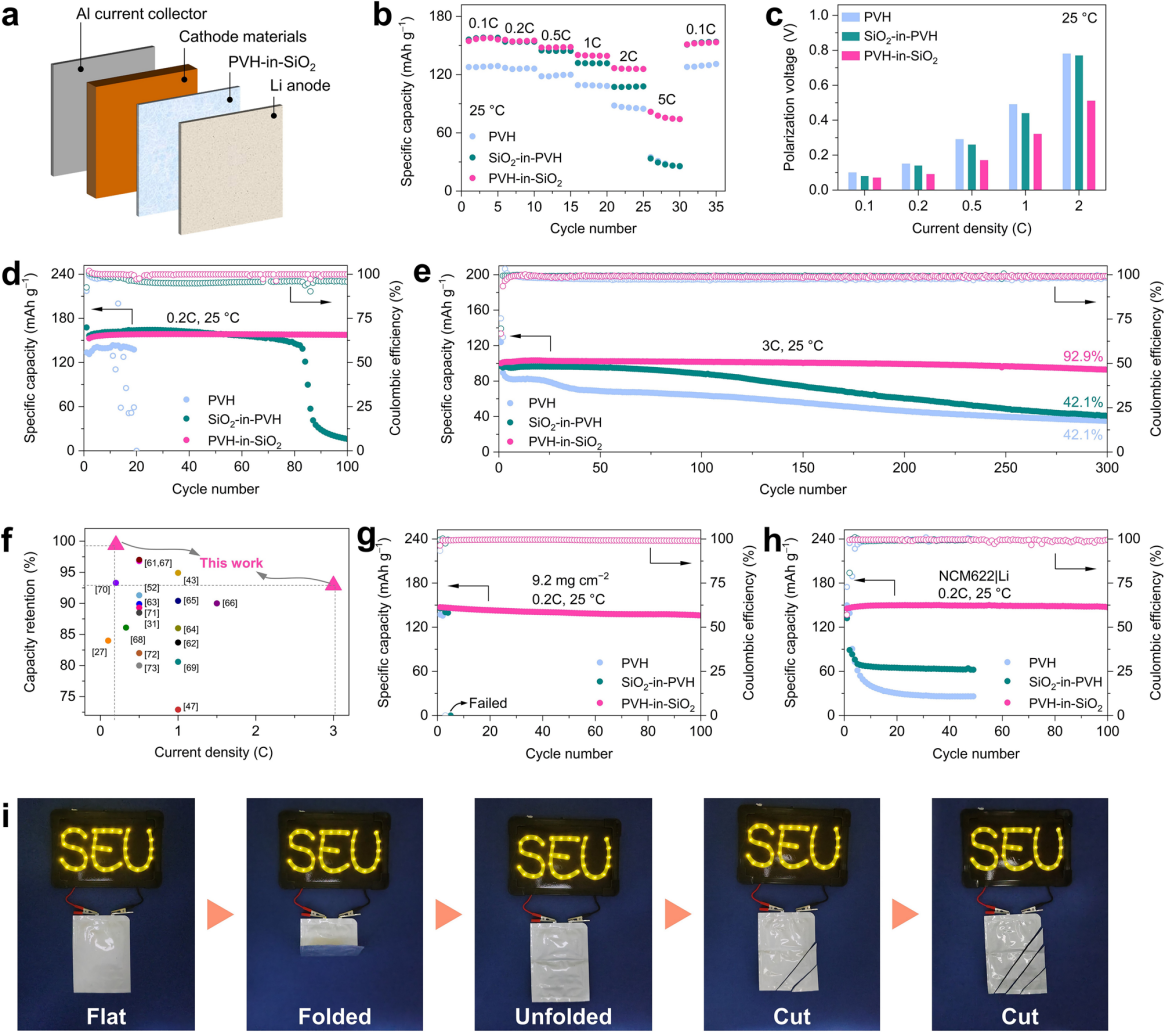
Electrochemical performance of solid-state full cells. **a** Schematic of the PVH-in-SiO_2_-based solid-state full cells. **b, c** Rate capability of LFP|PVH|Li, LFP|SiO_2_-in-PVH|Li, and LFP|PVH-in-SiO_2_|Li at 25 °C: **b** specific capacity and **c** corresponding polarization voltage at various current densities. The polarizations of LFP|PVH|Li and LFP|SiO_2_-in-PVH|Li at 5C are too large to determine their polarization voltages (Supplementary Fig. S17). **d, e** Cycling stability of LFP|PVH|Li, LFP|SiO_2_-in-PVH|Li, and LFP|PVH-in-SiO_2_|Li at the current density of **d** 0.2C and **e** 3.0C under 25 °C. **f** Comparison of specific capacity retention of LFP|PVH-in-SiO_2_|Li with recently reported solid-state LFP|Li batteries using polymer solid electrolytes. **g** Cycling performance of LFP|PVH-in-SiO_2_|Li at high mass loadings of 9.2 mg cm^−2^ under 25 °C (current density: 0.2C). **h** Cycling performance of NCM622|PVH-in-SiO_2_|Li at the current density of 0.2C under 25 °C. **i** Digital photographs of LED bulbs powered by LFP|PVH-in-SiO_2_|Li pouch cells under different states and conditions: flat, folded, unfolded, and cut

We first assembled solid-state full cells using LiFePO_4_ (LFP), PVH-in-SiO_2_, and Li metal as cathode, solid-state electrolyte, and anode, respectively (Fig. 4a). The mass loading of LFP is 1.5–2.5 mg cm^−2^. The rate performance was evaluated at various current densities under 25 °C (Figs. 4b and S23). The LFP|PVH-in-SiO_2_|Li full cells show high reversible specific capacities of 157.4, 154.5, 148.1, 139.6, and 126.0 mAh g^−1^ at 0.1C, 0.2C, 0.5C, 1C, and 2C, respectively. When the current density soars to 5C, a high specific capacity of 75.6 mAh g^−1^ is also achieved, which can immediately return to 152.6 mAh g^−1^ once the current density recovers to 0.1C. In contrast, the LFP|SiO_2_-in-PVH|Li full cells showcase much lower specific capacities at high current densities (e.g., only 27.4 mAh g^−1^ is delivered at 5C), despite that they have good specific capacities at small current densities (< 0.5C). In the case of LFP|PVH|Li, even worse performance is delivered at all current densities. These findings demonstrate that the PVH-in-SiO_2_ exhibits excellent rate capability, especially at high current densities, which can be further verified by the polarization voltage result (Figs. 4c and S24). For instance, the polarization voltage of LFP|PVH-in-SiO_2_|Li at 2C is approximately 0.51 V, which is only 65% and 66% of those of LFP|PVH|Li (0.78 V) and LFP|SiO_2_-in-PVH|Li (0.77 V).

The cycling performances of LFP|PVH-in-SiO_2_|Li full cells were also evaluated under 25 °C, as Fig. 4d shows. When the current density is fixed at 0.2C, the LFP|PVH-in-SiO_2_|Li delivers a high initial specific capacity of 152.8 mAh g^−1^, which gradually increases to 158.6 mAh g^−1^ after 20 cycles. The specific capacity increase is mainly attributed to the activation of LFP and the interface stabilization between electrodes and PVH-in-SiO_2_ [59, 60]. The specific capacity remains stable in the following cycles, and an outstanding capacity retention of 99.4% is obtained after 100 cycles. In addition, the LFP|PVH-in-SiO_2_|Li also exhibits a high average Coulombic efficiency of 99.8% upon cycling, indicating the excellent cyclability of PVH-in-SiO_2_. In contrast, both LFP|SiO_2_-in-PVH|Li and LFP|PVH|Li show poor cycling stability. The specific capacity of LFP|SiO_2_-in-PVH|Li quickly falls after 80 cycles, while the LFP|PVH|Li experiences abnormal discharging/charging behaviors after only 10 cycles and fails at the 20th cycle (Fig. S25). We further evaluated the long-term cyclability at a higher current density of 3C under 25 °C. The LFP|PVH-in-SiO_2_|Li delivers a high capacity retention of 92.9% after 300 cycles, whereas the LFP|SiO_2_-in-PVH|Li and LFP|PVH|Li only achieve a poor capacity retention of 42.1% (Fig. 4e). Furthermore, when we raise the current density to 5C, the LFP|PVH-in-SiO_2_|Li can still have an attractive capacity retention of 77.5% after 300 cycles at 25 °C (Fig. S26). Even compared to recently reported solid-state LFP|Li full cells, our LFP|PVH-in-SiO_2_|Li also shows overwhelming cycling stability, especially at high current densities (Fig. 4f and Table S4) [27, 31, 43, 47, 52, 61–73].

High-mass-loading LFP cathodes were also employed to demonstrate the potential of PVH-in-SiO_2_ in practical applications. Figure 4g shows the cycling performance of solid-state LFP|PVH-in-SiO_2_|Li full cells with a high mass loading of 9.2 mg cm^−2^ under 25 °C. An impressive specific capacity of 136.0 mAh g^−1^ is delivered at the current density of 0.2C after 100 cycles, corresponding to a superior capacity retention of 92.4%. In contrast, the LFP|SiO_2_-in-PVH|Li and LFP|PVH|Li full cells quickly fail after 5 and 3 cycles (Fig. S27). These findings verify that the PVH-in-SiO_2_ is promising for practical solid-state Li metal battery applications. In addition to the LFP cathode, we also employ LiNi_0.6_Co_0.2_Mn_0.2_O_2_ (labeled as NCM622) as a typical nickel-rich oxide cathode in order to investigate the compatibility of PVH-in-SiO_2_ with high-voltage cathode materials, as Fig. 4h displays. The resulted solid-state NCM622|PVH-in-SiO_2_|Li full cells exhibit a high initial specific capacity of 145.6 mAh g^−1^, which even slightly increases to 147.1 mAh g^−1^ after 100 cycles at the current density of 0.2C under 25 °C. On the contrary, the NCM622|SiO_2_-in-PVH|Li and NCM622|PVH|Li full cells quickly deteriorate in the initial several cycles and only deliver small specific capacities of 62.3 and 26.1 mAh g^−1^, respectively, after 50 cycles. These results suggest that the PVH-in-SiO_2_ holds excellent potential for high-energy–density solid-state Li metal batteries.

We also assembled LFP|PVH-in-SiO_2_|Li pouch cells with a lateral size of 3.5 × 4.0 cm^2^ to demonstrate the potential applications of our PVH-in-SiO_2_ for multipurpose solid-state batteries (Fig. 4i). The pouch cells can power 36 light-emitting diode (LED) bulbs under the flat state. When folded and unfolded in sequence, the pouch cells can still power the LED bulbs, implying that the PVH-in-SiO_2_ promises flexible solid-state Li metal batteries. Moreover, the pouch cells can still work properly without fuming or burning when they are cut into several small pieces, demonstrating their high safety under extremely harsh conditions.

### Versatility of Host–Guest Inversion Engineering Strategy

**Fig. 5:**
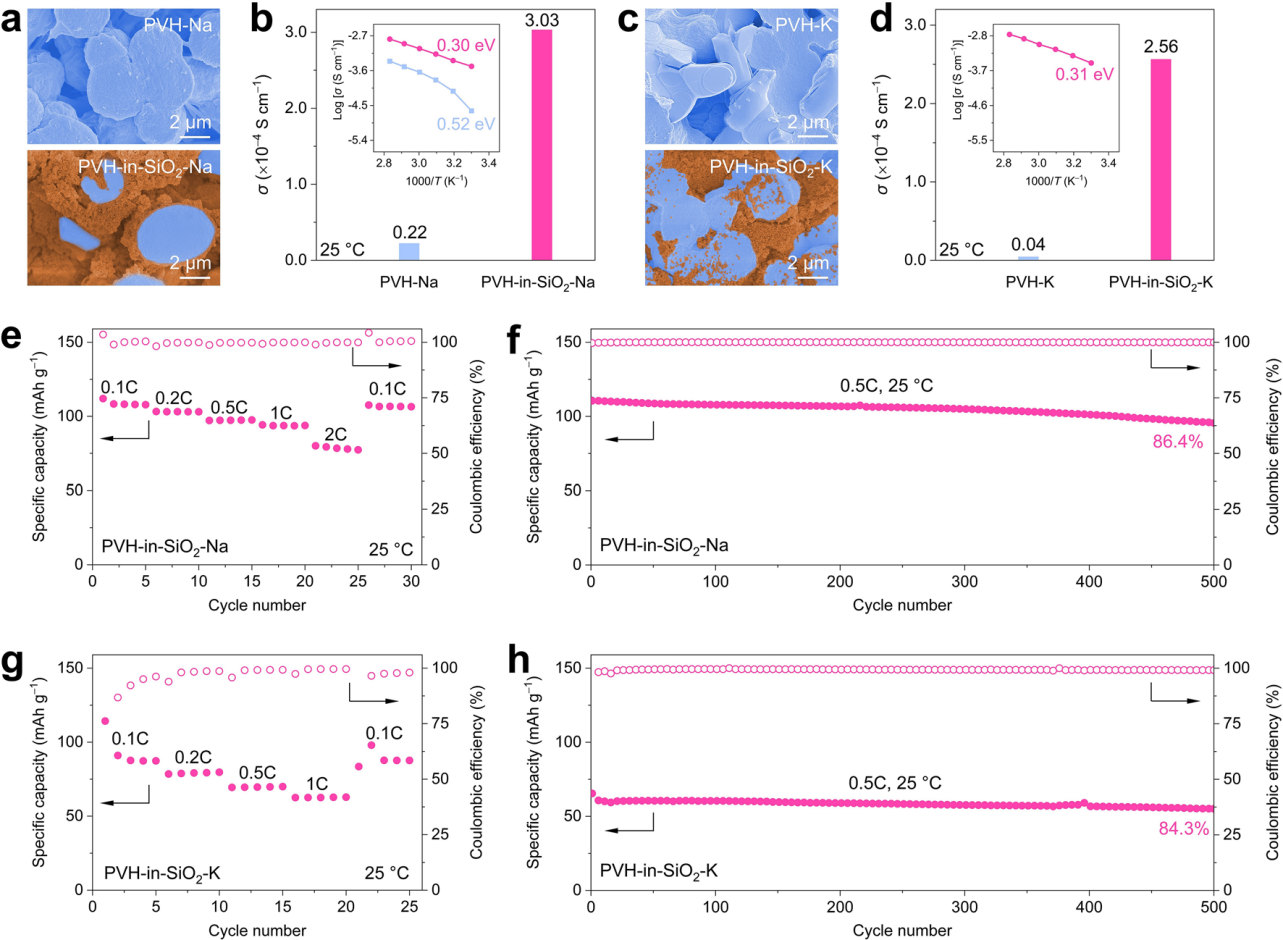
Morphologies, ion-conducting properties, and full-cell performance of PVH-in-SiO_2_-Na and PVH-in-SiO_2_-K. **a** SEM images of PVH-Na and PVH-in-SiO_2_-Na. **b** Ionic conductivities at 25 °C and activation energies (inset) of PVH-Na and PVH-in-SiO_2_-Na. **c** SEM images of PVH-K and PVH-in-SiO_2_-K. **d** Ionic conductivities at 25 °C and activation energies (inset) of PVH-K and PVH-in-SiO_2_-K. Note that the activation energy of PVH-K cannot be calculated (details in Supplementary information). **e**, **f** Electrochemical performance of NVP|PVH-Na|Na and NVP|PVH-in-SiO_2_-Na|Na full cells at 25 °C: **e** rate capability at various current densities and **f** cycling stability at 0.5C. **g**, **h** Electrochemical performance of KPB|PVH-K|K and KPB|PVH-in-SiO_2_-K|K full cells at 25 °C: **g** rate capability at various current densities and **h** cycling stability at 0.5C

The outstanding properties and performance of PVH-in-SiO_2_ for Li^+^ conduction and solid-state Li metal batteries motivate us to explore the feasibility of host–guest inversion engineering in solid-state Na and K metal batteries, which are promising for grid-scale energy storage because of their low costs and relatively high energy densities [74–76]. PVH-in-SiO_2_-Na and PVH-in-SiO_2_-K are obtained by simply replacing LiTFSI with NaTFSI and KTFSI, respectively, with other preparation parameters unchanged. Figure 5a depicts the SEM images of PVH-Na and PVH-in-SiO_2_-Na. The PVH also forms interconnected spheres, which are also densely covered by SiO_2_ nanoparticles. As a result, the PVH-in-SiO_2_-Na exhibits a high ionic conductivity of 3.03 × 10^−4^ S cm^−1^ at 25 °C, which is almost 14 times that of PVH-Na (2.2 × 10^−5^ S cm^−1^, Figs. 5b and S28). In addition, PVH-in-SiO_2_-Na also shows a low activation energy of 0.30 eV, significantly smaller than that of PVH-Na (0.52 eV, inset of Figs. 5b and S29). Similar results are also observed in the case of PVH-K and PVH-in-SiO_2_-K (Fig. 5c). Specifically, the PVH-in-SiO_2_-K exhibits a high ionic conductivity of 2.56 × 10^−4^ S cm^−1^ at 25 °C, which is 64 times that of PVH -K (4.0 × 10^−6^ S cm^−1^, Figs. 5d and S30). In addition, the PVH-in-SiO_2_-K also delivers a low activation energy of 0.31 eV (inset of Fig. 5d), whereas we cannot obtain reliable activation energy for PVH-K owing to their extremely low ionic conductivities and abnormal EIS curves (Figs. S31 and S32). These findings once again demonstrate that the host–guest inversion engineering strategy is a versatile approach to significantly facilitating ion transport for achieving high room-temperature ionic conductivity.

To evaluate the implementations of PVH-in-SiO_2_-Na and PVH-in-SiO_2_-K, solid-state full cells were assembled using Na_3_V_2_(PO_4_)_3_ (NVP) and potassium Prussian blue (KPB) as cathode materials, respectively. In the case of NVP|PVH-in-SiO_2_-Na|Na (Fig. 5e), high specific capacities of 108.1, 103.1, 97.3, and 93.7 mAh g^−1^ are delivered at the current density of 0.1C, 0.2C, 0.5C, and 1C under 25 °C, respectively. Even when the current density is further increased to 2C, a large specific capacity of 78.4 mAh g^−1^ is retained, demonstrating the remarkable rate capability of the PVH-in-SiO_2_-Na. The cycling stability of NVP|PVH-in-SiO_2_-Na|Na was assessed at the current density of 0.5C under 25 °C (Fig. 5f). An excellent specific capacity of 95.5 mAh g^−1^ is achieved after 500 cycles, corresponding to a capacity retention of as high as 86.4%. Furthermore, the average Coulombic efficiency upon cycling exceeds 99.9%, indicating the superb cyclability of PVH-in-SiO_2_-Na. On the contrary, the NVP|PVH-Na|Na full cells quickly fail after several cycles, verifying their inferior rate and cycling performance (Figs. S33 and S34). Similar results are also observed in the cases of PVH-in-SiO_2_-K and PVH-K (Fig. 5g, h). The KPB|PVH-in-SiO_2_-K|K delivers fantastic rate capability (87.7 and 62.6 mAh g^−1^ at 0.1C and 1C, respectively) and cycling performance (84.3% capacity retention after 500 cycles at 0.5C) under 25 °C. In contrast, the KPB|PVH-K|K cannot work at all. These electrochemical performance results undoubtedly confirm the versatility and superiority of our host–guest inversion engineering strategy, making it promising for next-generation, advanced solid-state alkali metal batteries.

## Conclusions

We have successfully realized a superionic CSE using SiO_2_ nanoparticles as cost-effective passive ceramic hosts and the interconnected PVH spheres as polymer guests, forming an innovative “PVH-in-SiO_2_” (i.e., “polymer guest-in-ceramic host”) architecture with optimized and continuous SiO_2_/PVH interfacial contacts. The PVH-in-SiO_2_ architecture not only significantly inhibits the crystallization of PVH to offer sufficient amorphous areas for Li^+^ transport, but also greatly facilitates the dissociation of LiTFSI to achieve high-concentration moveable Li^+^. Owing to the low diffusion energy barriers and high diffusion coefficient, these moveable Li^+^ are thermodynamically and kinetically favorable to migrate to and transport at the continuous SiO_2_/PVH interfaces. Therefore, the PVH-in-SiO_2_ exhibits an overwhelmingly high ionic conductivity of 1.32 × 10^−3^ S cm^−1^ at 25 °C, with an ultralow residual solvent content of 2.9 wt%. The PVH-in-SiO_2_ also shows outstanding electrochemical stability with Li metal anode and various cathode materials. As a result, the LFP|PVH-in-SiO_2_|Li full cells deliver excellent rate capability and cyclability at high current rates (92.9% and 77.5% capacity retentions at 3C and 5C after 300 cycles under 25 °C, respectively). In addition, the PVH-in-SiO_2_ showcases good compatibility with high-mass-loading cathodes and high-capacity NCM622, demonstrating good cycling and safety performance. More significantly, the versatility of our host–guest inversion engineering strategy is verified by constructing advanced PVH-in-SiO_2_-Na and PVH-in-SiO_2_-K by simply replacing LiTFSI with NaTFSI and KTFSI, respectively. The PVH-in-SiO_2_-Na and PVH-in-SiO_2_-K not only have high ionic conductivities at 25 °C (both are in the order 10^−4^ S cm^−1^) but also exhibit excellent full-cell performance. Our host–guest inversion engineering strategy offers a simple and large-scale approach to fabricating superionic CSEs with high-throughput Li^+^ transport and low costs, paving an avenue for the practical application of next-generation solid-state Li metal batteries and beyond.

## Supplementary Information

Below is the link to the electronic supplementary material.

Supplementary file1 (DOCX 11474 KB)
