# Supplementary material for: Host–Guest Inversion Engineering Induced Superionic Composite Solid Electrolytes for High-Rate Solid-State Alkali Metal Batteries
Source: Nanomicro Lett. 2025 Mar 17;17:190. doi: 10.1007/s40820-025-01691-7 (PMC11911308; doi:10.1007/s40820-025-01691-7)
Supplement: Supplementary file 1 — Supplementary file1 (DOCX 11474 KB) [file 40820_2025_1691_MOESM1_ESM.docx]

Supporting Information for

**Host-Guest Inversion Engineering Induced Superionic Composite Solid Electrolytes for High-Rate Solid-State Alkali Metal Batteries**

Xiong Xiong Liu^1,#^, Long Pan^1,#,^*, Haotian Zhang^1^, Pengcheng Yuan^1^, Mufan Cao^1^, Yaping Wang^1^, Zeyuan Xu^1^, Min Gao^1^, Zheng Ming Sun^1,^*

^1^School of Materials Science and Engineering, Southeast University, Nanjing 211189, P. R. China

^#^ Xiong Xiong Liu and Long Pan contributed equally to this work.

* Corresponding authors. E-mail: [zmsun@seu.edu.cn](mailto:zmsun@seu.edu.cn) (Zheng Ming Sun); [panlong@seu.edu.cn](mailto:panlong@seu.edu.cn) (Long Pan)

**S1 Methods**

***Synthesis of SiO_2_ nanoparticles*** [S1]. For the synthesis of 158 nm SiO_2_ nanoparticles, 225 mL of anhydrous ethanol (AR, ≥ 99.7%, Sinopharm), 30 mL of distilled water, and 5 mL of ammonium hydroxide solution (25–28%, Macklin) were mixed and stirred for 30 min, to which 15 mL of tetraethyl orthosilicate (TEOS, 99%, Aladdin) was added in three times (*i*.*e*., 5 mL per time) under stirring. The obtained solution was then kept stirred for 2 h at ambient temperature. After reaction, the white precipitate was filtrated, washed with anhydrous ethanol three times, and dried at 80 °C overnight under vacuum in sequences, obtaining the 158 nm SiO_2_ nanoparticles. The synthesis of 488 nm SiO_2_ nanoparticles is similar to the above one, except that the feed ratios and reaction time are somewhat different. Specifically, 81 mL of anhydrous ethanol and 24 mL of ammonium hydroxide solution were mixed and stirred for 30 min, to which 4.2 mL of TEOS was added under stirring. The solution was subsequently kept stirred for 1 h at room temperature, obtaining white precipitate. The collection, washing, and drying processes of the white precipitate were the same to the previous procedures.

***Preparation of PVH,*** ***SiO_2_-in-PVH, and PVH-in-SiO_2_ CSEs.*** The CSE films were prepared by a simple solution-cast method. Typically, 0.6 g of PVH (average *M_w_* = 455000, Macklin) and 0.6 g of LiTFSI (99.9%, Macklin) were added in 6 mL of *N*,*N*-dimethylformamide (DMF, AR, 99.5%, Macklin), which was kept stirred until the PVH and LiTFSI were dissolved to form a transparent solution. Subsequently, different weight ratios of SiO_2_ nanoparticles were added (*i*.*e*., SiO_2_/PVH = 0/100, 20/100, and 70/100), followed by stirring for another 12 h. The obtained homogeneous dispersion was cast into a petri dish, which was initially dried in an air circulation oven at 30 °C to remove most of the DMF solvent and then dried in a vacuum oven at 120 °C for 24 h. After the temperature cooled down, a white film was obtained, followed by storing in an Ar-filled glove box for further investigations. The films with SiO_2_/PVH weight ratio of 0/100, 20/100, and 70/100 were denoted as PVH, SiO_2_-in-PVH, and PVH-in-SiO_2_, respectively. The preparation of PVH-in-SiO_2_-Na and PVH-in-SiO_2_-K films is same to that of PVH-in-SiO_2_, except that the LiTFSI was replaced by NaTFSI and KTFSI, respectively.

***Material Characterization.*** XRD patterns were recorded by a Haoyuan DX-2700BH diffractometer (Cu Kα, λ = 1.5418 Å). SEM images were acquired by a FEI Sirion field emission scanning electron microscope. Thermogravimetric (TG) curves were recorded under nitrogen atmosphere from ambient temperature to 800 °C with a ramping rate of 10 ℃/min. Raman spectra were collected by a WITec Alpha 300 Access spectrometer. SSNMR spectra were recorded by an Agilent 600 DD2 spectrometer with a magnetic field strength of 14.1 T at the resonance frequency of 233.08 MHz. Dipole decoupling magic-angle spinning (DD/MAS) was employed during the acquisition process. The samples for SSNMR experiment were placed in a pencil-type zirconia rotor with a 4.0 mm outside diameter. The rotor was spun at the speed of 8 kHz with a recycle delay of 5 s. The chemical shift was referenced to LiCl.

***Electrochemical tests.*** All cells were assembled using CR2032-type coin cells unless otherwise specified. The ionic conductivity was calculated based on EIS profiles, which were recorded at the frequency range from 10^6^ to 10^−1^ Hz and an applied amplitude of 5 mV using symmetric cells with stainless steel (SS) electrode (*viz*., SS|CSEs|SS). The electrochemical stability was confirmed by LSV at the scanning rate of 0.1 mV s^−1^ from 1.0 to 6.0 V using asymmetric SS|CSEs|Li cells. The Li^+^ transference number was tested by applying a 10 mV direct current (DC) polarization voltage on symmetric Li|CSEs|Li cells. The EIS, LSV, and DC polarization were conducted using a BioLogic SP-150 electrochemical station. GCD profiles of symmetric cells were recorded by LAND CT2001A and NEWARE MIHW-200-160CH battery testers at various current densities. All measurements were conducted at 25 °C unless otherwise noted. All cells were rested overnight before testing.

***Full cell assembly.*** The cathode material (LFP, NCM622, NVP, or KPB) was mixed with conductive additive (super P) and binder (poly (vinylidene difluoride)) at a weight ratio of 80:10:10. The mixture was dispersed in *N*-methylpyrrolidone (NMP) to form a homogenous slurry, which was coated on Al foils, followed by a vacuum-drying at 120 °C for 12 h. The mass loadings of LFP, NCM622, NVP, and KPB are 1.5–2.5, 2.5–3.5, 2.5–3.5, and 1.0–1.5 mg cm^−2^, respectively. Note that the high-mass-loading LFP cathodes (8.0–11.0 mg cm^−2^) were also prepared using the same approach, except that the thickness of the coated slurry on Al foils were different. These cathodes were paired with different alkali metal anode to assemble full cells, which were tested on a NEWARE MIHW-200-160CH battery tester. Note that 5 μL of liquid electrolyte (1M LiPF_6_ in EC: DEC= 1:1 vol % for LFP|Li, 1M NaClO_4_ in PC: FEC= 95:5 vol % for NVP|Na, and 4M KFSI in EC: DEC= 1:1 vol % for KPB|K) was added on the cathode surface, improving its contact with CSEs and facilitating the Li^+^ diffusion in cathodes. The galvanostatic discharging/charging (GCD) tests were carried out with a voltage range of 2.8–4.2 V for LFP|Li full cells, 3.0–4.3 V for NCM622|Li full cells, 2.2–3.8 V for NVP|Na full cells, and 2.0–4.25 V for KPB|K full cells. All cells were rested overnight before testing. All electrochemical tests were conducted under 25 °C.

***DFT calculations.*** DFT calculations were performed by the Vienna Ab Initio Package (VASP) within the generalized gradient approximation (GGA) using the Perdew, Burke, and Enzerhof (PBE) formulation. [S2-S4] The ionic cores were described by projected augmented wave (PAW) potentials and the valence electrons were taken into account employing a plane wave basis set with a kinetic energy cutoff of 450 eV. [S5, S6] The Gaussian smearing method with a width of 0.05 eV was utilized to allow for partial occupancies of the Kohn-Sham orbitals. The electronic energy was considered self-consistent when the energy change was smaller than 10^−4^ eV. A geometry optimization was considered convergent when the force change was smaller than 0.05 eV/Å. The dispersion interactions were described using Grimme's DFT-D3 methodology. The equilibrium lattice constants of structures were optimized when using a 1×1×1 Monkhorst-Pack k-point grid for Brillouin zone sampling. The Li ions migration barriers in the SiO_2_ Slab, SiO_2_-SiO_2_, PVH + LiTFSI with SiO_2_ slab structures were calculated by employing the Climbing Image-Nudged Elastic Band methods. In addition, the nudged elastic band (NEB) method and the dimer method are employed to calculate the dissociation energy of LiTFSI. In the NEB method, the path between the reactant and product is discretized into a series of structural images. The image that is closest to a likely transition state structure was then employed as an initial guess structure for the dimer method.

***MD simulations*.** MD simulations were conducted using the Gromacs 2019.6. [S7] The simulation setups involved the uniform blending of 373 Li^+^ ions and 373 TFSI^–^ ions with 100 PVH chains (each PVH chain was composed of five vinylidene fluoride units and five hexafluoropropylene units). In addition, 52 and 182 SiO_2_ nanospheres (with a diameter of 0.8 nm) were incorporated to simulate the PVH-in-SiO_2_ and SiO_2_-in-PVH systems, respectively. The GAFF force field [S8] and RESP atomic charges [S9] were utilized to model PVH and TFSI^–^, which were derived via the Multiwfn program by fitting them to the electrostatic potential calculated at the B3LYP-D3/def2TZVP level of theory in Gaussian 16. Parameters proposed by Semino [S10] and Heinz [S11] et al. were employed to describe Li^+^ and SiO_2_ nanospheres, respectively. The Lennard Jones (LJ) potential with a cut-off value of 1.2 nm was employed to describe van der Waals interactions. Lorentz-Berthelot mixing rules were used to obtain the LJ parameters between different atom pairs. Electrostatic interactions within a short range were truncated at 1.2 nm, while the long-range interactions were calculated utilizing the particle-mesh Ewald method. All initial configurations were energy-minimized, followed by equilibration at 298.15 K and 1 bar for 5 ns. Finally, 20-ns production runs were carried out under the NPT ensemble (298.15 K, 1 bar) with a time step of 1 fs. Equations of motion were integrated by the leap-frog algorithm. Three-dimensional periodic boundary conditions were applied throughout simulations.

***Finite element analysis.*** COMSOL Multiphysics 6.2 software was used for the simulation. The current physics field of the AC/DC module was employed to conduct the simulation analysis. Geometry creation is the basic step in building a model. It involves defining the physical shape and spatial layout of the model. According to the complexity of the model, the two-dimensional space is selected. In the current physics field, the upper boundary potential is set to 1mV, the lower boundary is grounded, and the current is conserved in different regions. By simulating and analyzing several different structures, the electric field distribution on the electrode surface is obtained. The formulas are as follows:

$$\text{∇}\text{ ∙}\text{ }\text{J}\text{=}\text{ }\text{Q}_{\text{j, υ}}$$

$$\text{J}\text{ = }\text{σ}\text{ ∙}\text{E}\text{+ }\text{J}_{\text{e}}$$

$$\text{E}\text{ = -∆}\text{V}$$

where, $\text{J}$ is the current density (mA cm^−2^), $\text{Q}_{\text{j, υ}}$ is the charge density (C m^−3^), σ is the ionic conductivity (S m^−1^), $\text{E}$ is the electric field intensity (V m^−1^), and $\text{V}$ is the applied potential (V).

**S2 Supplementary Figures and Tables**

**Table S1** Densities of active ceramics, passive ceramics, and PVH

| **Type** | **Substances** | **Density (g cm^−3^)** | **Refs.** |
| --- | --- | --- | --- |
| Active ceramics | Li_7_La_3_Zr_2_O_12_ (LLZO) | 5.10 | [S12, S13] |
|  | Li_0.33_La_0.57_TiO_3_ (LLTO) | 5.04 | [S14, S15] |
|  | Li_1+_*_x_*Al*_x_*Ge_2−_*_x_*(PO_4_)_3_ (LAGP) | 3.56 | [S16-S19] |
|  | Li_1.3_Al_0.3_Ti_1.7_(PO_4_)_3_ (LATP) | 2.94 | [S20-S22] |
|  | Li_10_GeP_2_S_12_ (LGPS) | 1.99 | [S23] |
|  | LiPSCl | 1.81 | [S24, S25] |
|  | Li_3_YCl_6_ | 2.45 | [S26] |
| Passive ceramics | SiO_2_ | 2.20 | [S27, S28] |
|  | Al_2_O_3_ | 3.40–4.00 | [S29, S30] |
|  | TiO_2_ | 3.90–4.30 | [S31, S32] |
|  | MgO | 3.60 | [S33, S34] |
|  | ZnO | 5.60 | [S35, S36] |
| Polymer | PVH | 1.76–1.80 | [S37] |

The density of SiO_2_ is approaching to that of PVH, indicating that they occupy similar volumes when they have the same weight. Consequently, we can achieve an innovative "polymer guest-in-ceramic host" (*i*.*e*., PVH-in-SiO_2_) architecture with minimized SiO_2_/PVH weight ratio when compared to other active ceramics such as LLZO and LATP (see examples in **Fig. S1a,** **b**).


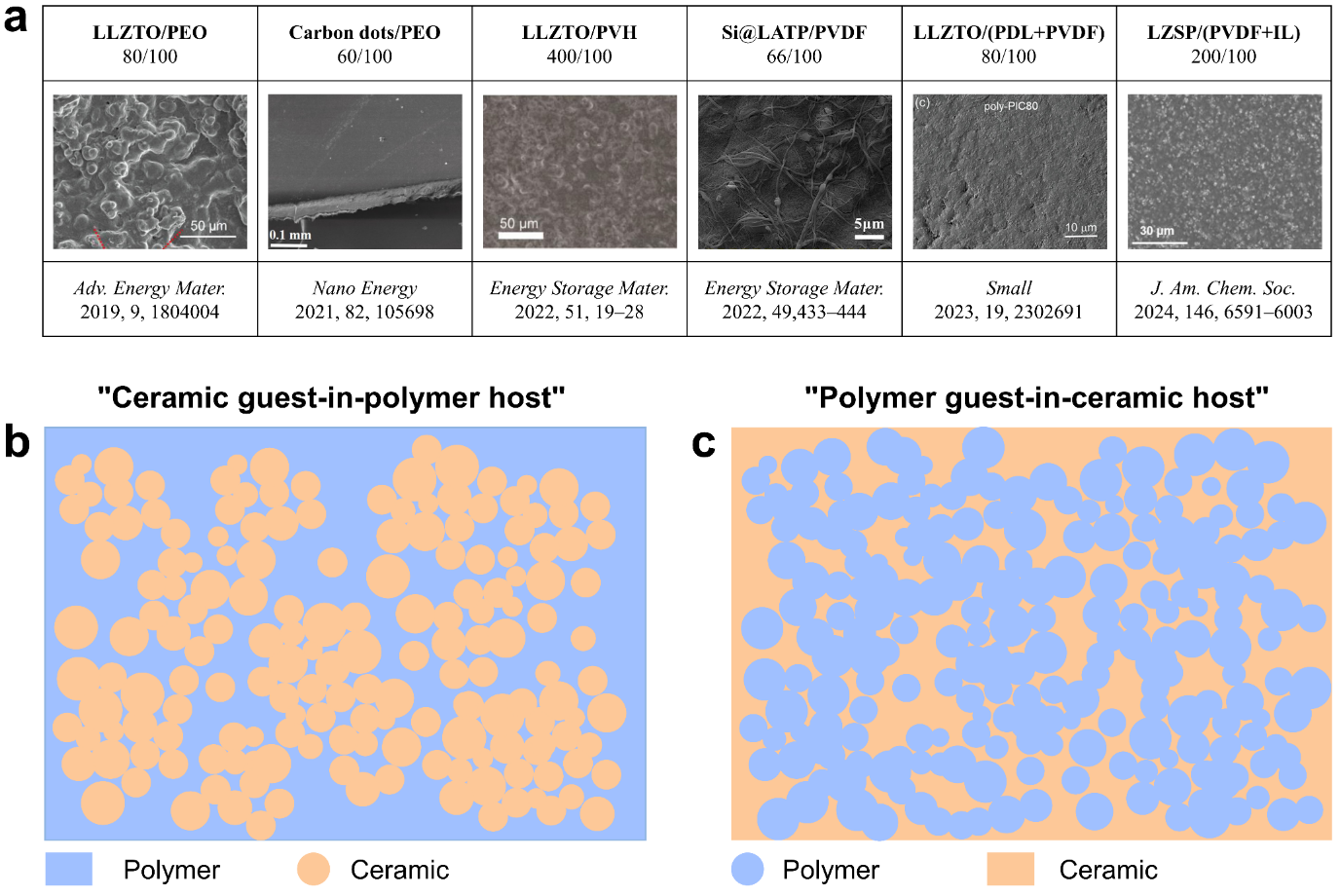


**Fig. S1** (**a**) SEM images of previously reported CSEs based on active ceramics, exhibiting traditional "ceramic guest-in-polymer host" architectures. [S38-S43] (**b, c**) Illustration of morphology comparison of (**b**) traditional "ceramic guest-in-polymer host" and (**c**) our innovative "polymer guest-in-ceramic host" architectures. **a** were adapted with permissions S38-S43]

It is worth pointing out that many papers proposed high ceramic/polymer weight ratios (up to 400/100 wt%) to increase interfacial contacts. Although they used the term "polymer-in-ceramic" in their papers, the ceramics were still dispersed in polymer matrices (**Fig. S1a**), forming traditional "ceramic guest-in-polymer host" architectures (**Fig. S1b**) instead of the "polymer guest-in-ceramic host" (**Fig. S1c**).

**Table S2** Comparison of ionic conductivity (at 25 °C) and residual solvent content of our PVH-in-SiO_2_ with previously reported CSEs and polymer solid-state electrolytes

| Solid-state electrolytes | Ionic conductivity  (mS cm^−1^) | Residual solvent  (wt%) | Refs |
| --- | --- | --- | --- |
| PVH-in-SiO_2_ | **1.32×10^−3^** | **2.9** | **This work** |
| BTO/LLTO/PVDF | 8.2×10^−4^ | 14.4 | [S44] |
| Defective PVH | 7.84×10^−4^ | 17.6 | [S45] |
| 2D SiO_2_/PVH | 3.7×10^−4^ | 1.5 | [S46] |
| NaNbO_3_/PVDF | 5.56×10^−4^ | 14.3 | [S47] |
| d-HNTs/PVDF | 2.9×10^−4^ | 3.95 | [S48] |
| Si_3_N_4_/PVDF | 5.7×10^−4^ | 12.9 | [S49] |
| PbZr_x_Ti_1−x_O_3_/PVDF | 1.16×10^−4^ | 7.9 | [S50] |
| LATP/PVDF | 6.0×10^−4^ | 16.6 | [S51] |
| PVDF | 1.18×10^−4^ | 15.0 | [S52] |
| PVH | 1.2×10^−4^ | 13.0 | [S53] |
| POE-F | 1.7×10^−4^ | 8.8 | [S54] |
| PVDF-LPPO | 4.84×10^−4^ | 18.9 | [S55] |
| P(VDF-TrFE-CTFE) | 3.1×10^−4^ | 9.9 | [S56] |
| PCL-MDI-DBNPG | 2.2×10^−4^ | 3.8 | [S57] |
| LLZTO/PVDF | 1.2×10^−4^ | 7.0 | [S58] |
| Molecular sieves/PVDF | 4.5×10^−4^ | 17.4 | [S59] |
| CuPcLi/PVT | 8.0×10^−4^ | 10.0 | [S60] |


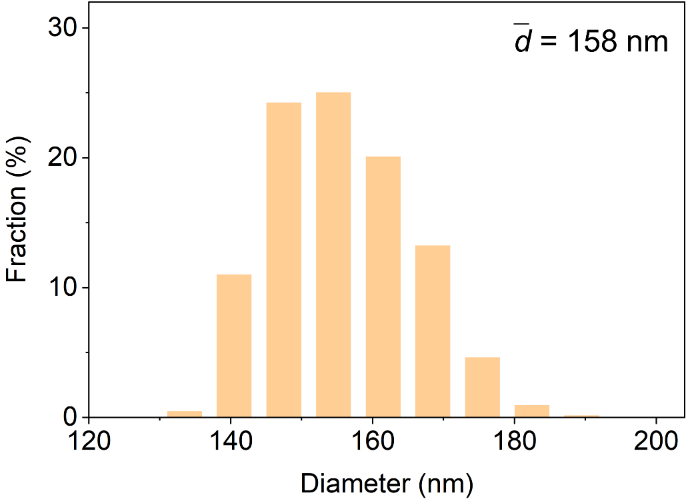


**Fig. S2** Diameter statistics diagram of the SiO_2_ nanoparticles in **Fig. 2c**


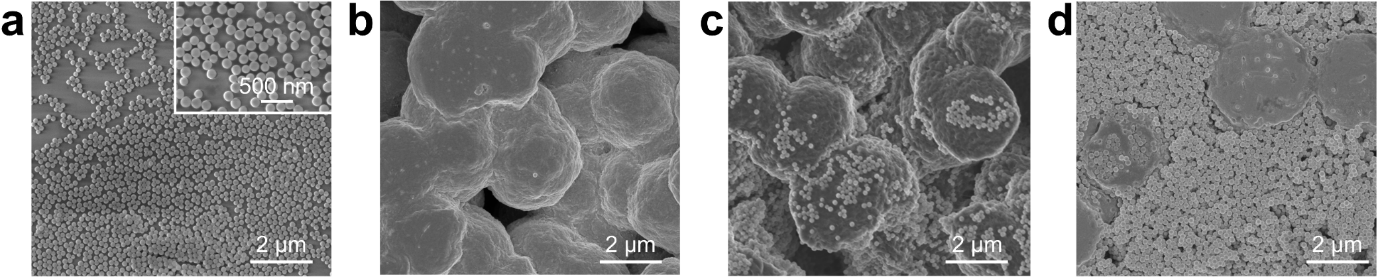


**Fig. S3** Unprocessed SEM images of **Fig. 2c–f**: (**a**) SEM images of SiO_2_ nanoparticles. b–d, Top-view SEM images of (**b**) PVH, (**c**) SiO_2_-in-PVH, and (**d**) PVH-in-SiO_2_ CSEs


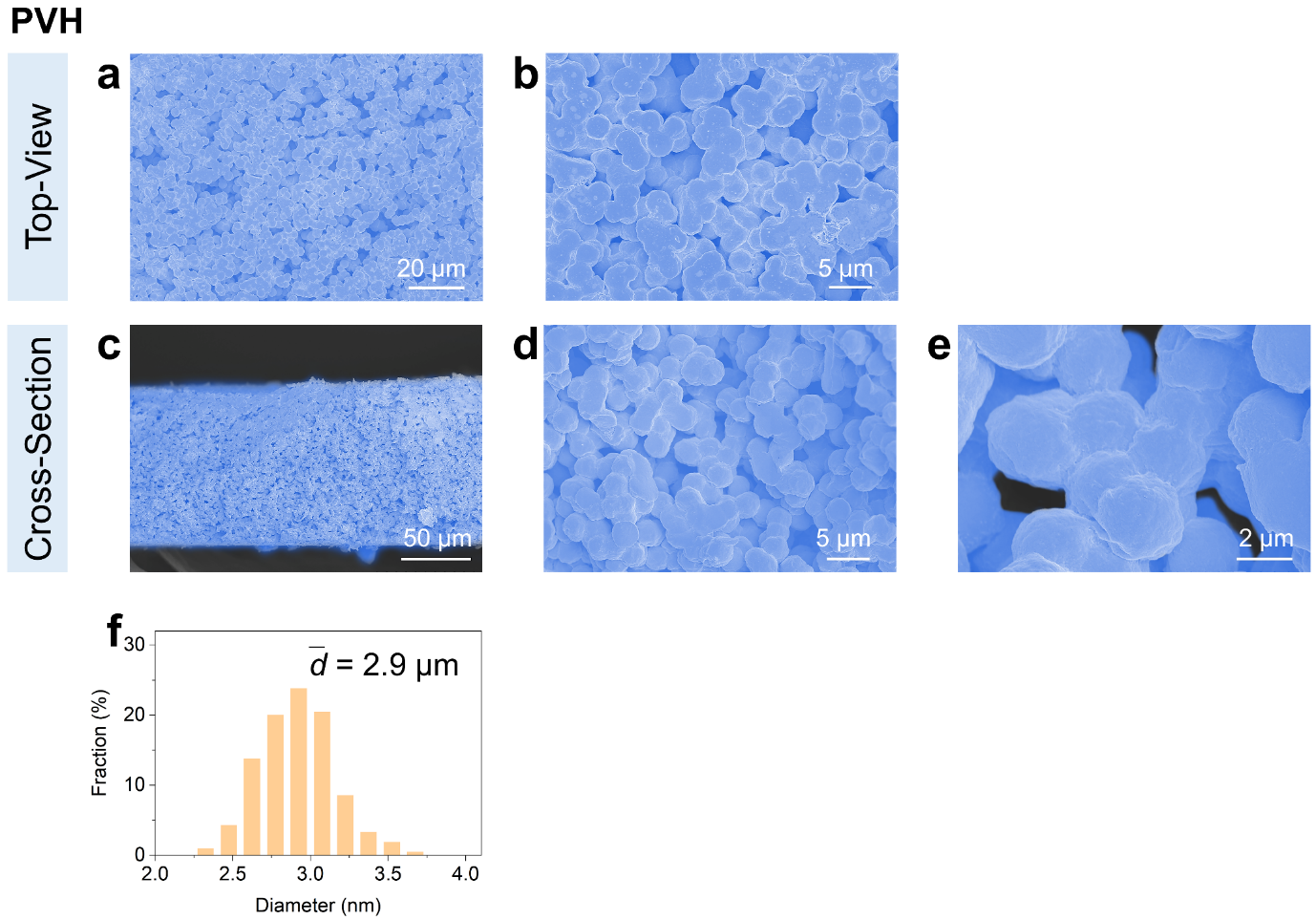


**Fig. S4** (**a, b**) Top-view and (**c–e**) cross-sectional SEM images of PVH. (f) Diameter statistics diagram of the PVH spheres. These SEM images are supplementary to that in **Fig. 2d**, further demonstrating the interconnected sphere morphology of PVH and the existence of voids


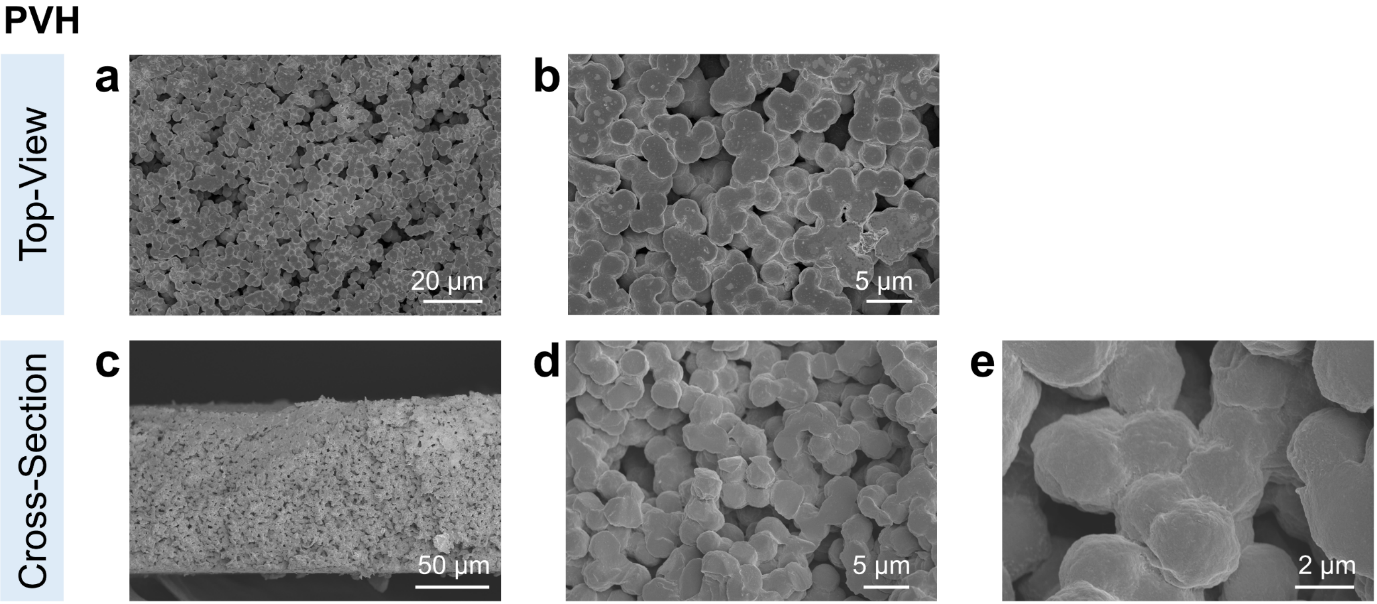


**Fig. S5** Unprocessed SEM images of **Fig. S4**: (**a, b**) Top-view and (**c–e**) cross-sectional SEM images of PVH


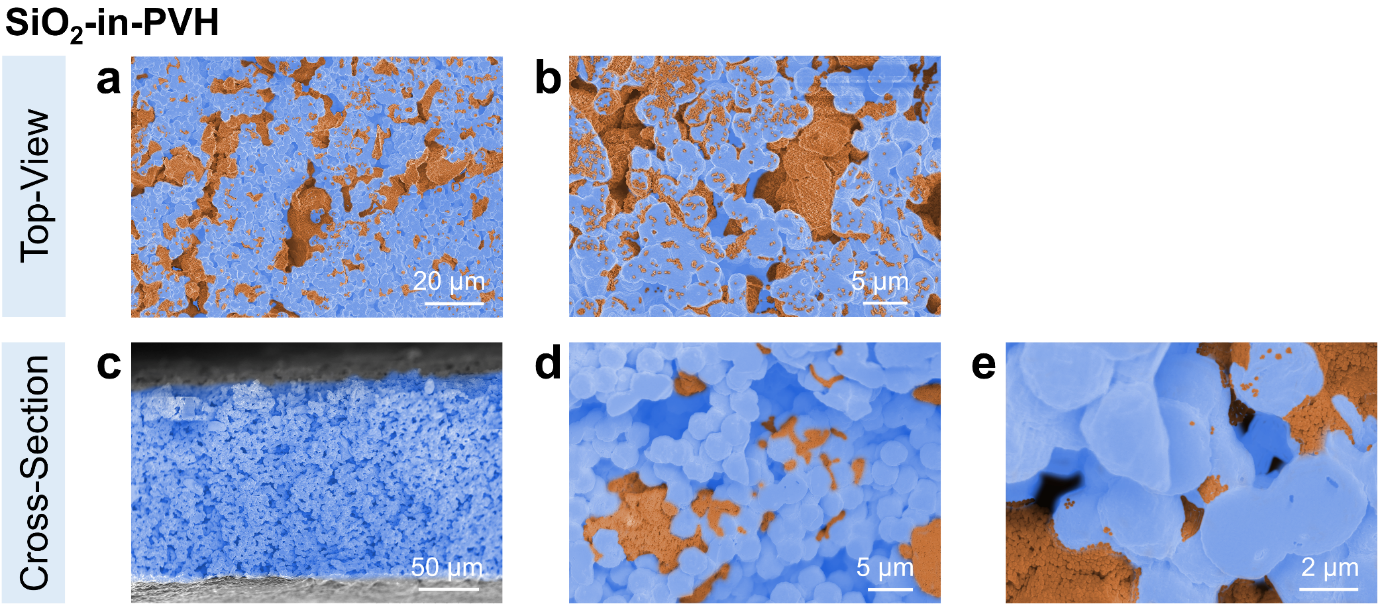


**Fig. S6** (**a, b**) Top-view and (**c–e**) cross-sectional SEM images of SiO_2_-in-PVH. These SEM images are supplementary to that in **Fig. 2e**. These images further demonstrate the formation of SiO_2_-in-PVH morphology, because 1) the voids in the PVH matrix are unevenly and partially filled by SiO_2_ nanoparticles, and 2) the PVH spheres are loosely covered by SiO_2_ nanoparticles


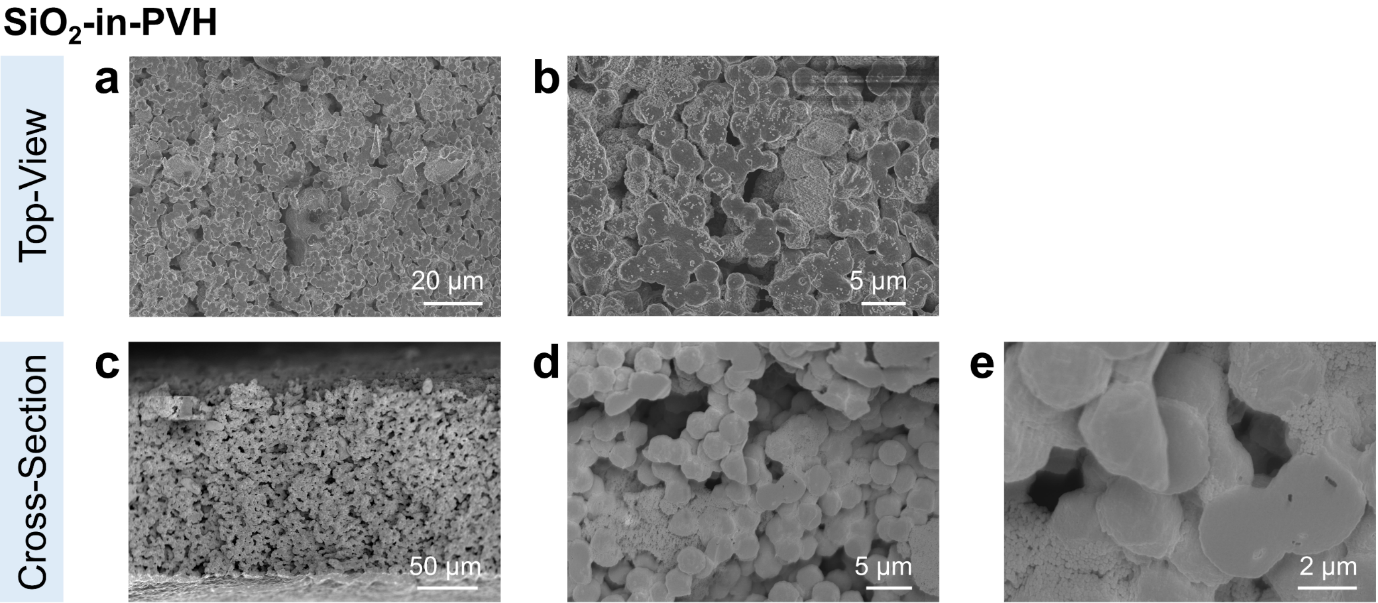


**Fig. S7** Unprocessed SEM images of **Fig. S6**: (**a, b**) Top-view and (**c–e**) cross-sectional SEM images of SiO_2_-in-PVH


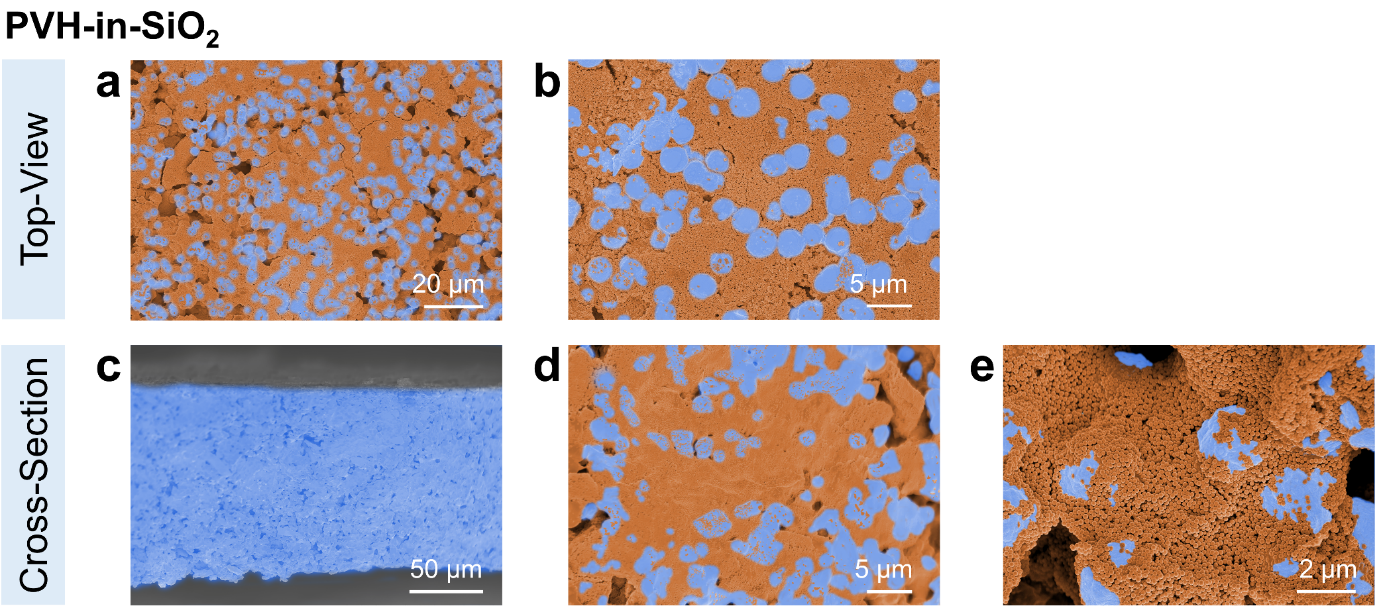


**Fig. S8** (**a, b**) Top-view and (**c–e**) cross-sectional SEM images of PVH-in-SiO_2_. These SEM images are supplementary to that in **Fig. 2f**. These images further demonstrate that the SiO_2_ nanoparticles fully fill the voids and cover the PVH spheres, maximizing the interfacial contact between PVH and SiO_2_ nanoparticles and forming interconnected interfacial highways for Li^+^ conducting


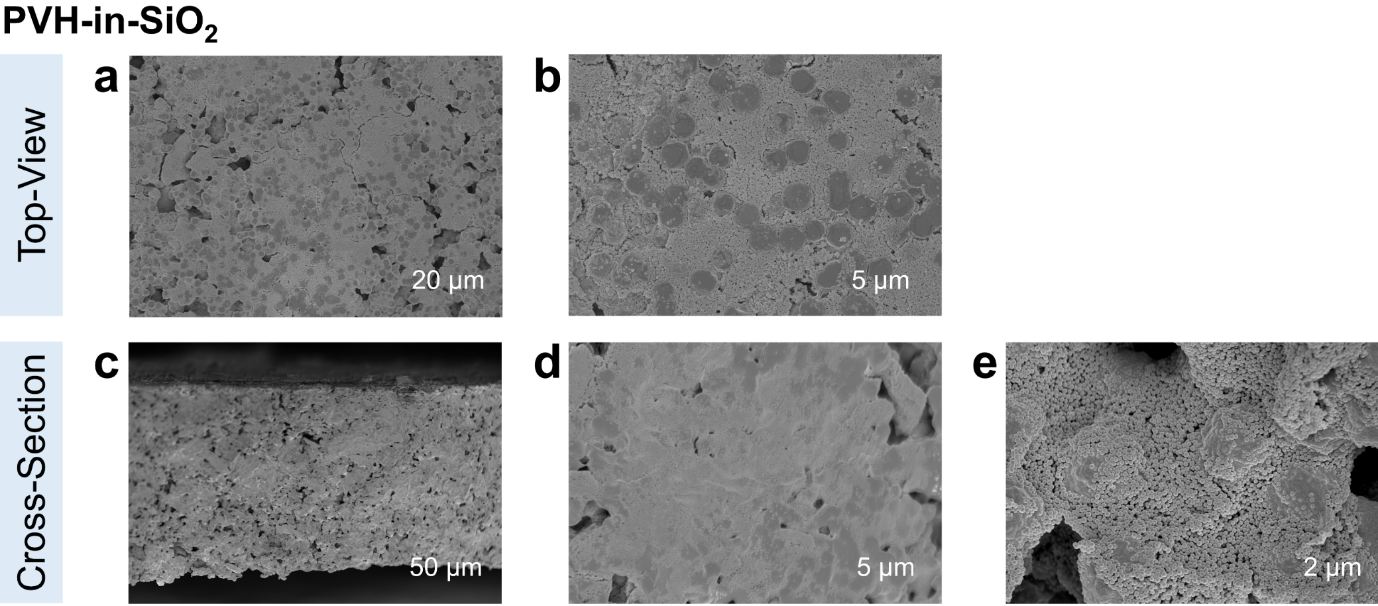


**Fig. S9** Unprocessed SEM images of **Fig. S8**: (**a, b**) Top-view and (**c–e**) cross-sectional SEM images of PVH-in-SiO_2_


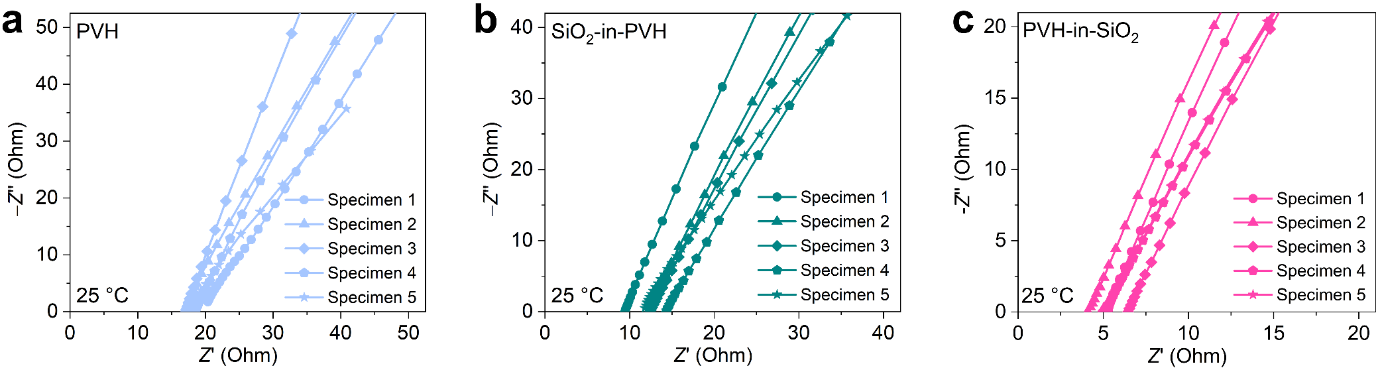


**Fig. S10** EIS curves of SS|CSEs|SS symmetric cells using (a) PVH, (b) SiO_2_-in-PVH, and (c) PVH-in-SiO_2_ as CSEs at 25 °C. For each sample, five cells (*i*.*e*., specimens) were assembled and tested. The corresponding ionic conductivities were calculated and plotted in **Fig. 2g**


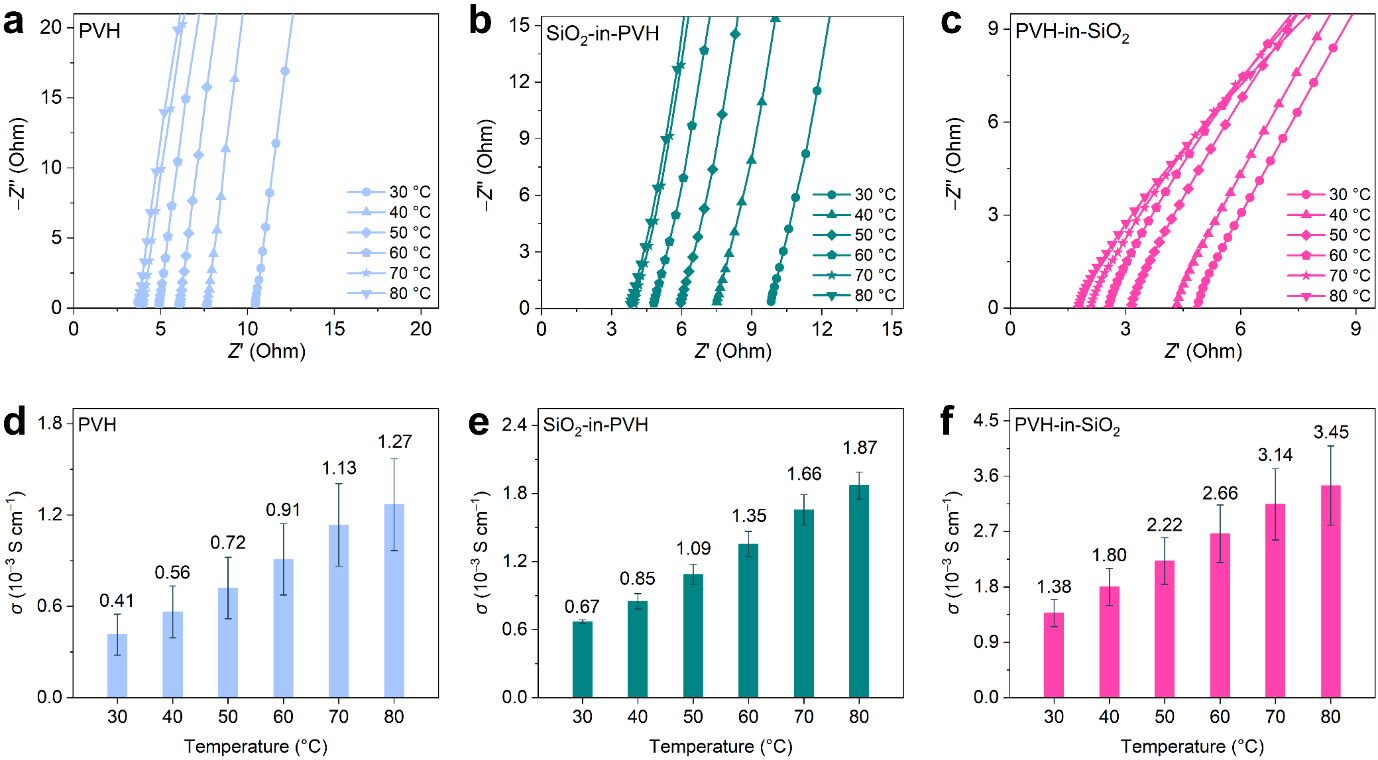


**Fig. S11** (**a–c**) Typical EIS curves of SS|CSEs|SS symmetric cells using (**a**) PVH, (b) SiO_2_-in-PVH, and (**c**) PVH-in-SiO_2_ as CSEs at different temperatures. Note that five cells (*i*.*e*., specimens) were assembled and tested for each sample, but only one EIS curve was plotted for each sample because of the limited layout. (**d–f**) Ionic conductivities (*σ*) of (d) PVH, (e) SiO_2_-in-PVH, and (**f**) PVH-in-SiO_2_ at different temperatures, which were calculated from the corresponding EIS curves


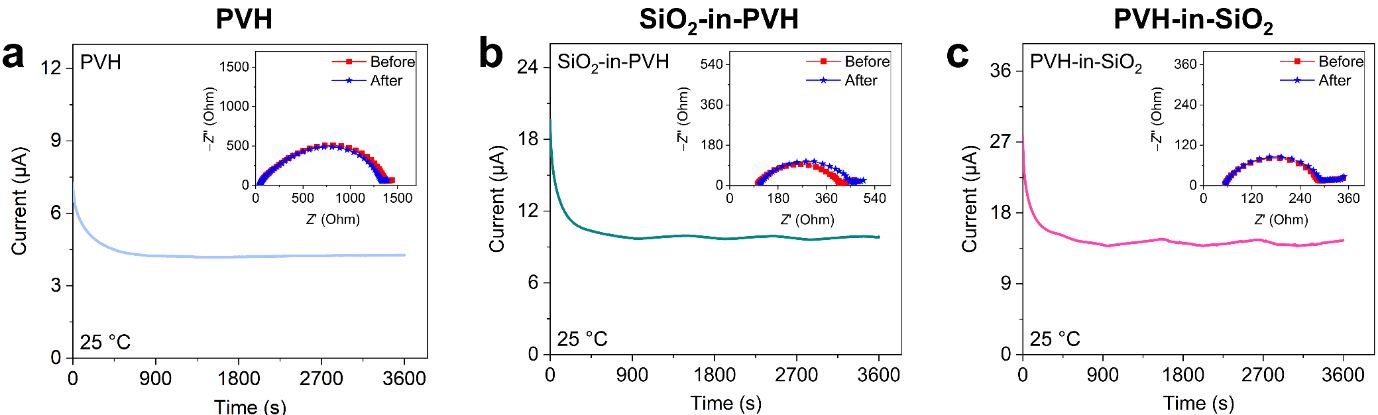


**Fig. S12** Typical DC polarization curves of Li|Li symmetric cells using (**a**) PVH, (**b**) SiO_2_-in-PVH, and (**c**) PVH-in-SiO_2_ as CSEs. The insets are the corresponding EIS curves before and after the DC polarization. All measurements were conducted with a DC polarization voltage of 10 mV and at 25 °C. Note that five cells (*i*.*e*., specimens) were assembled and tested for each sample, but only one DC polarization curve and corresponding EIS curves were plotted for each sample because of the limited layout. The corresponding Li^+^ transference numbers were calculated and plotted in **Fig. 2i**

Note that the Li^+^ transference number was calculated by the Bruce-Vincent-Evans equation of *t* = $\frac{\text{I}_{\text{s}}\text{(∆}\text{V}\text{-}\text{I}_{\text{0}}\text{R}_{\text{0}}\text{)}}{\text{I}_{\text{0}}\text{(∆}\text{V}{\text{-}\text{I}}_{\text{s}}\text{R}_{\text{s}}\text{)}}$, where *I*_0_ and *I*_s_ stand for the initial and steady-state direct current polarizations, respectively; *∆V* represents the applied polarization voltage (10 mV); *R*_0_ and *R*_s_ indicate the impedance of Li metal surface passivation layer before and after polarization.

The EIS profiles before and after polarization were fitted using the following equivalent circuit. *R*_b_ represents the impedance of CSE; *R*_int_ denotes the interfaces impedance between the PVH and SiO_2_, and *R*_ct_ represents the impedance of charge transfer at the CSE (*i*.*e*., PVH, SiO_2_-in-PVH, and PVH-in SiO_2_) and electrode (*i*.*e*., Li metal surface) interfaces. *R*_ct_ truly reflects the impedance changes of passivation layer on the lithium metal surface. Therefore, *R*_0_ and *R*_s_ in the Bruce-Vincent-Evans equation are equal to *R*_ct_ before and after polarization, respectively.


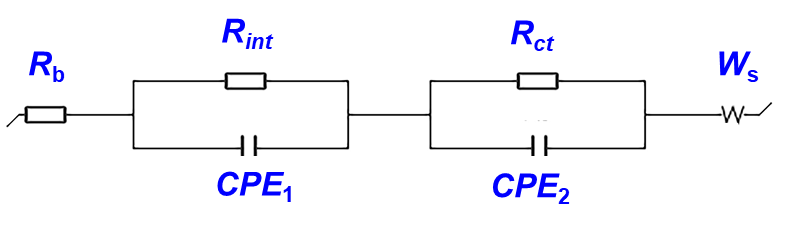


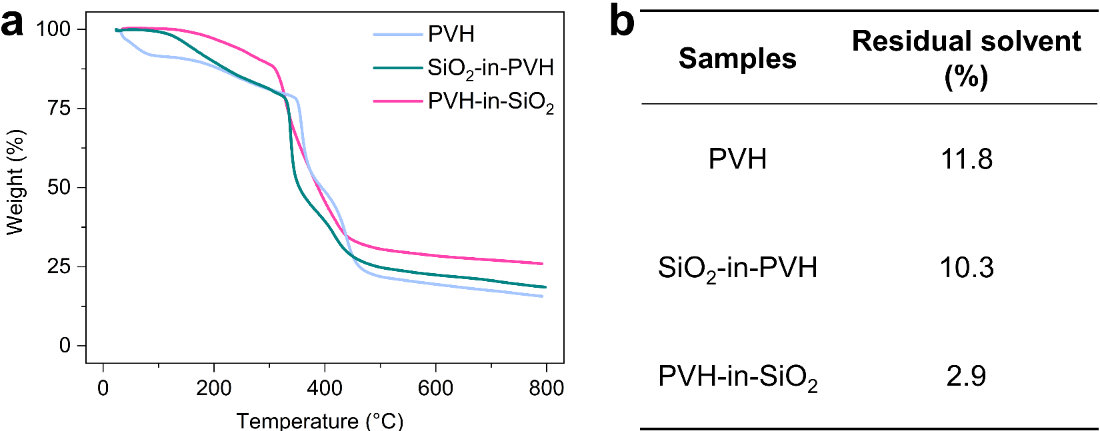


**Fig. S13** (**a**) TG curves and (**b**) corresponding residual solvent contents of PVH, SiO_2_-in-PVH, and PVH-in-SiO_2_. The residual solvent contents were calculated based on the total weight loss from ambient temperature to 200 °C

Note that the weight loss below 100 °C is attributed to the trapped moisture evaporation, which is mainly caused by the high hydroscopicity of LiTFSI. The trapped moisture content is significantly reduced from 8.5 wt% (PVH) to approximately 0 wt% (SiO_2_-in-PVH and PVH-in-SiO_2_), implying that the introduction of SiO_2_ nanoparticles can dramatically inhibit the moisture adsorption.


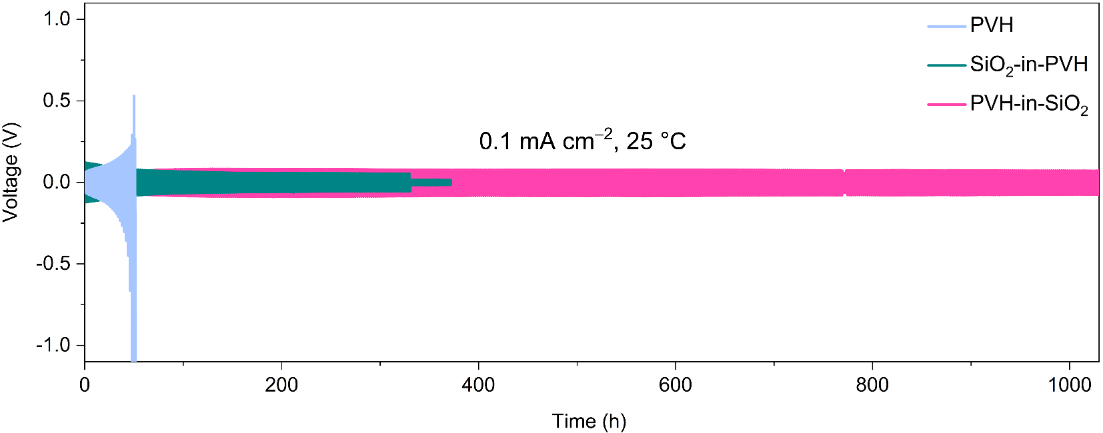


**Fig. S14** Galvanostatic voltage profiles of Li|Li symmetric cells at 0.1 mA cm^−2^ (1 h per step) of PVH, SiO_2_-in-PVH, and PVH-in-SiO_2_. The measurements were carried out at 25 °C

**Table S3** Comparison of activation energy, high-voltage stability, and Li symmetric cells total capacity of our PVH-in-SiO_2_ with other reported CSEs and polymer solid-state electrolytes.

| Solid-state electrolytes | Activation energy (eV) | High-voltage stability (V) | Li symmetric cells  (Total capacity) | Refs. |
| --- | --- | --- | --- | --- |
| PVH-in-SiO_2_ | **0.17** | **5.00** | **200 mAh cm^−2^** | **This work** |
| BTO/LLTO/PVDF | 0.20 | 4.8 | 180 mAh cm^−2^ | [S44] |
| Defective PVH | 0.21 | 4.4 | 120 mAh cm^−2^ | [S45] |
| 2D SiO_2_/PVH | 0.30 | 4.78 | 100 mAh cm^−2^ | [S46] |
| NaNbO_3_/PVDF | 0.22 | 4.7 | 280 mAh cm^−2^ | [S47] |
| d-HNTs/PVDF | 0.21 | 5.0 | 200 mAh cm^−2^ | [S48] |
| Si_3_N_4_/PVDF | 0.21 | 4.8 | 280 mAh cm^−2^ | [S49] |
| PbZr_x_Ti_1−x_O_3_/PVDF | 0.318 | 4.58 | 190 mAh cm^−2^ | [S50] |
| LATP/PVDF | 0.213 | 4.5 | 260 mAh cm^−2^ | [S51] |
| PVDF | 0.23 | 4.65 | 200 mAh cm^−2^ | [S52] |
| PVH | 0.17 | 4.7 | 120 mAh cm^−2^ | [S53] |
| POE-F | / | 5.0 | 112 mAh cm^−2^ | [S54] |
| PVDF-LPPO | 0.22 | 4.8 | 140 mAh cm^−2^ | [S55] |
| P(VDF-TrFE-CTFE) | 0.26 | 4.6 | 60 mAh cm^−2^ | [S56] |
| LLZTO/PVDF | 0.33 | 3.9 | 59 mAh cm^−2^ | [S58] |


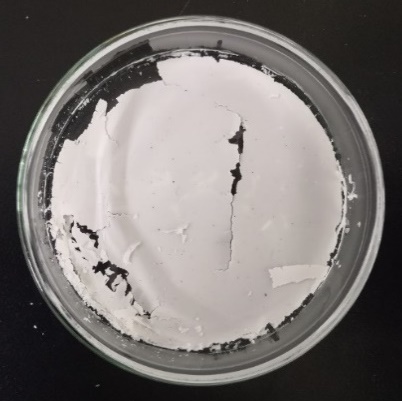


**Fig. S15** Photograph of an as-prepared CSE film with the SiO_2_/PVH weight ratio of 100/100 wt%


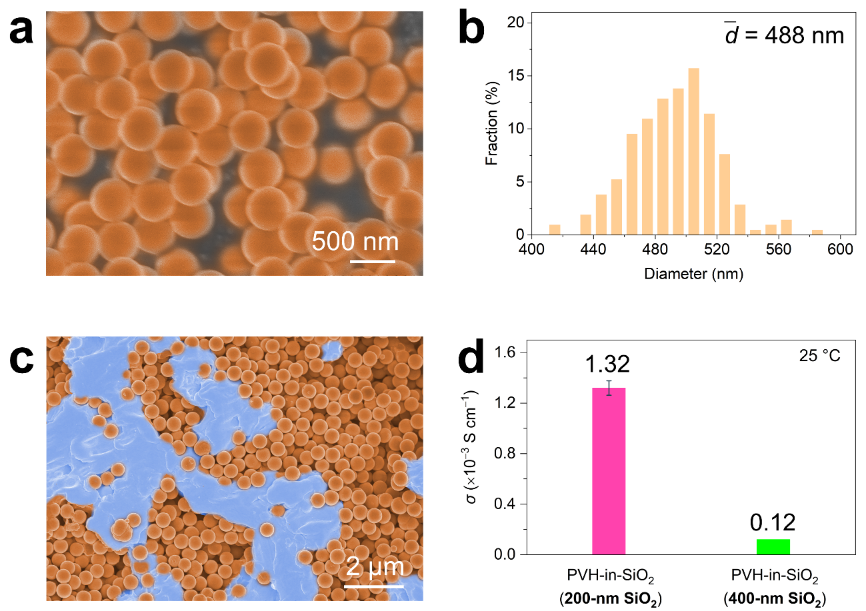


**Fig. S16** (**a**) SEM image and (**b**) the corresponding diameter statistics diagram of the 488 nm SiO_2_ nanoparticles. (**c**) Top-view SEM image of PVH-in-SiO_2_ that was prepared using the 488 nm SiO_2_ nanoparticles. (**d**) Comparison of ionic conductivities (*σ*) of PVH-in-SiO_2_ using different sized SiO_2_ nanoparticles

Introducing the 488 nm SiO_2_ nanoparticles can also result in the PVH-in-SiO_2_ architecture. However, the interconnected sphere morphology of the PVH matrix is destroyed. In addition, the increase of the SiO_2_ nanoparticle diameter also leads to the decrease of SiO_2_/PVH contact area. Therefore, the ionic conductivity of PVH-in-SiO_2_ using the 488 nm SiO_2_ nanoparticles is significantly smaller than that using the 158 nm SiO_2_ nanoparticles. (The film thickness for ionic conductivity tests is approximately 120–150 µm.)


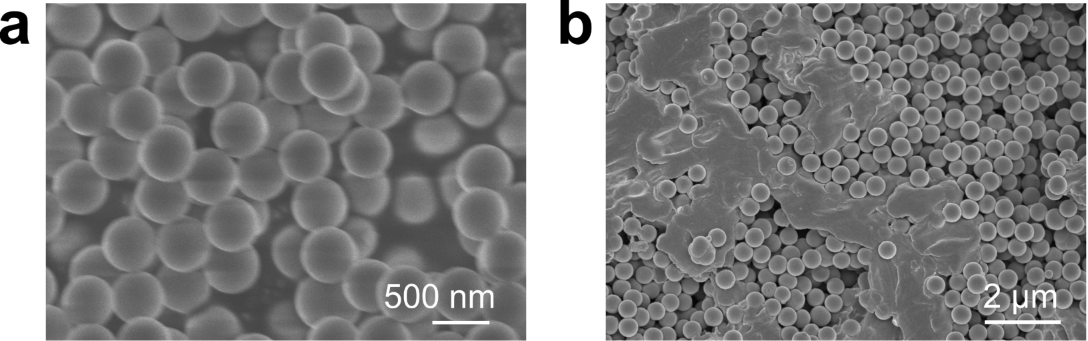


**Fig. S17** Unprocessed SEM images of **Fig. S16 a,** **c**: (**a**) SEM image of the 488 nm SiO_2_ nanoparticles. (**b**) Top-view SEM image of PVH-in-SiO_2_ that was prepared using the 488 nm SiO_2_ nanoparticles


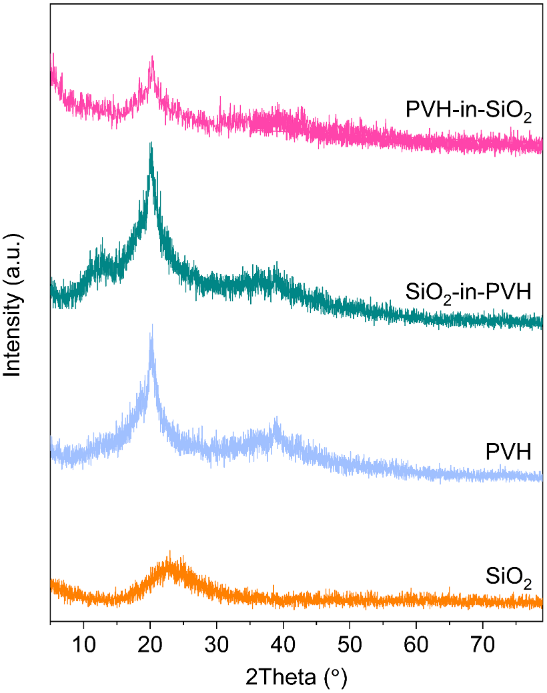


**Fig. S18** XRD patterns of SiO_2_, PVH, SiO_2_-in-PVH, and PVH-in-SiO_2_

The crystallinities in the PVH, SiO_2_-in-PVH, and PVH-in-SiO_2_ were calculated using the following formula:

$\begin{aligned} \text{Crystallinity}\text{ }\text{=}\text{ }\frac{\text{Area of crystalline peaks}}{\text{Area of all peaks }\left( \text{crystalline}\text{ }\text{+}\text{ }\text{amorp}\text{h}\text{ous} \right)}\text{ }\text{×}\text{ }\text{100\%}\# \end{aligned}$

Peak areas were obtained using the peak analyzer function in an OriginPro project (Learning Edition, copyright © 1991-2023 OriginLab Corporation). The peak area data are presented in the table below:

| **Samples** | **Area of crystalline peaks** | **Area of all peaks** | **Crystallinity** |
| --- | --- | --- | --- |
| PVH | 85 | 307 | 28% |
| SiO_2_-in-PVH | 92 | 375 | 25% |
| PVH-in-SiO_2_ | 58 | 309 | 19% |


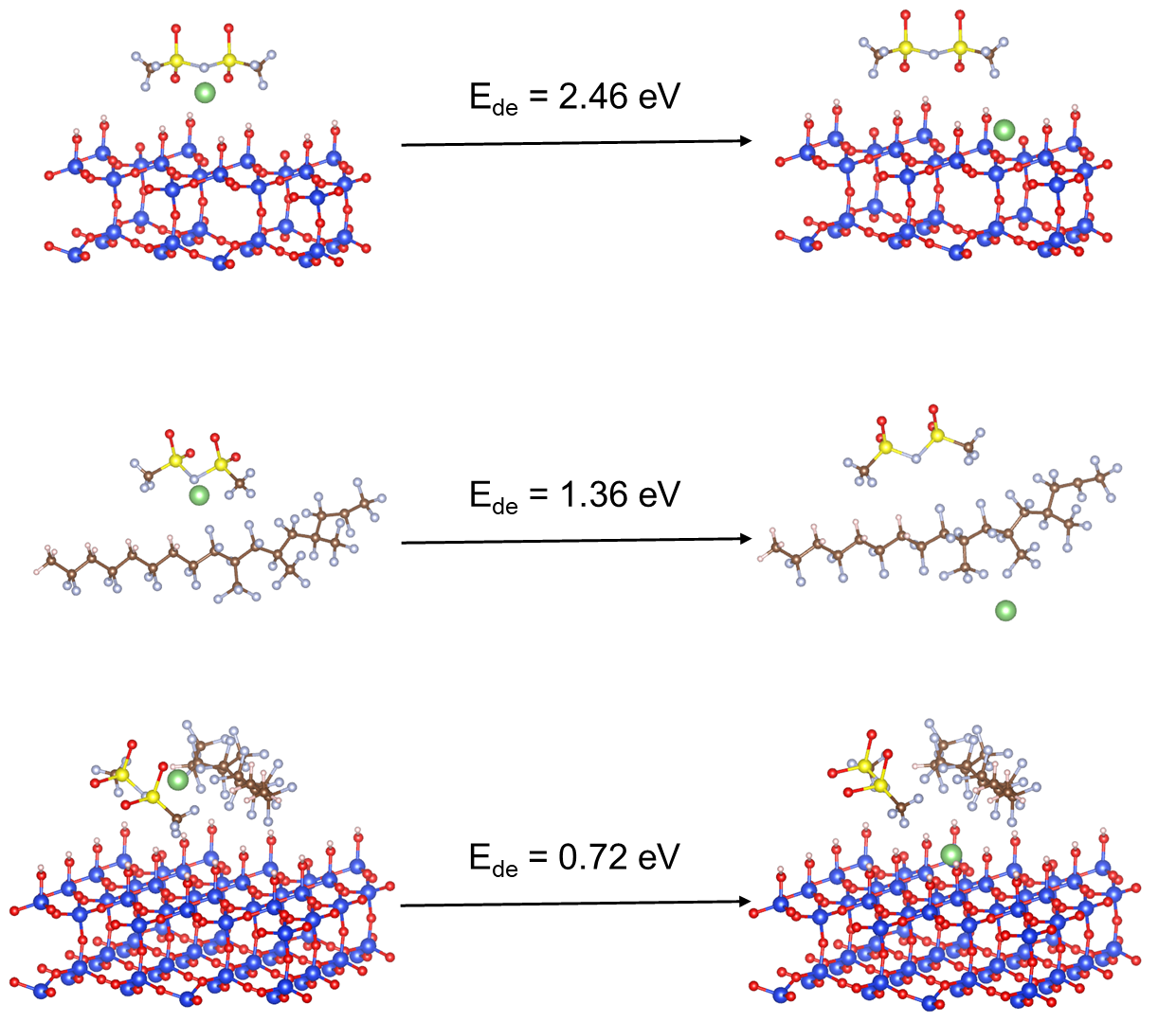


**Fig. S19** Dissociation energy of LiTFSI on SiO_2_ surface, PVH chains, and SiO_2_/PVH interface determined by DFT calculations


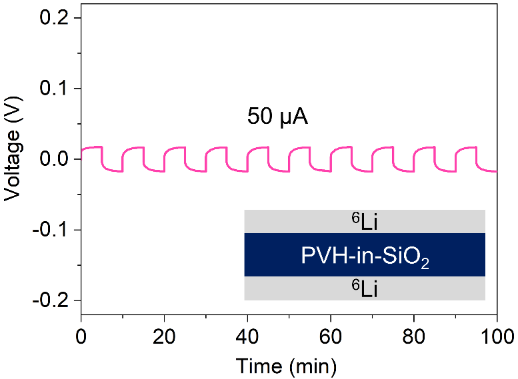


**Fig. S20** Li striping/plating curves of the ^6^Li symmetric cells using PVH-in-SiO_2_ as CSEs. The ^6^Li symmetric cells were tested at the current of 50 µA for 10 cycles, and the striping and plating time for each cycle were 5 min. The inset is the schematic of the ^6^Li symmetric cells, and the diameter of the ^6^Li foils is 12 mm


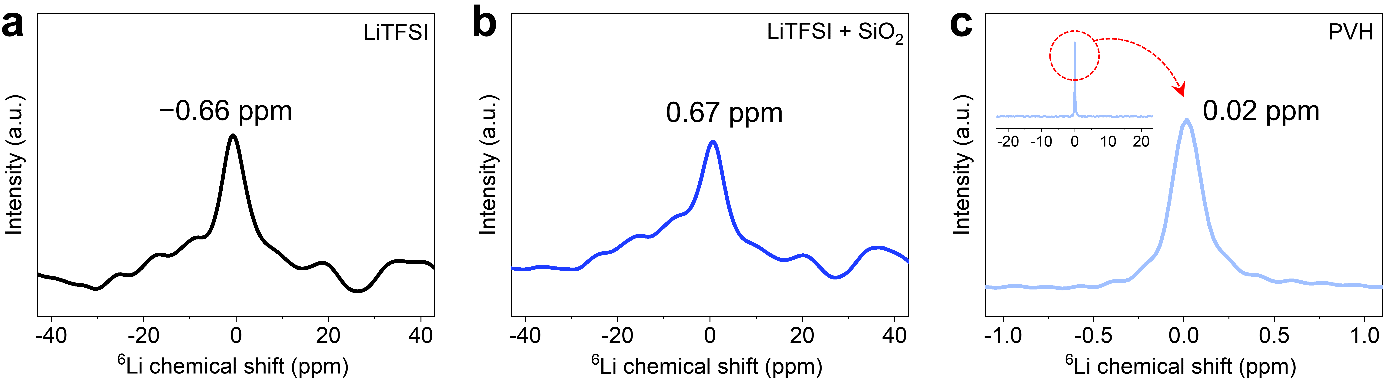


**Fig. S21** ^6^Li SSNMR spectra of pristine (**a**) LiTFSI, (**b**) LiTFSI + SiO_2_, and (**c**) PVH. Note that the sample "LiTFSI + SiO_2_" was prepared by mixing LiTFSI and SiO_2_ nanoparticles in solvent (DMF), followed by a vacuum-drying process

In the case of PVH (**Fig. S21c**), there is one chemical environment for ^6^Li^+^, which is reflected by the intensive and symmetrical peak located at 0.02 ppm. This result is very similar to that in the case of PVH-in-SiO_2_ (*i*.*e*., the curve before cycling in **Fig. 3d**), indicating that most of the Li^+^ in the PVH-in-SiO_2_ before cycling are located in the PVH matrix instead of at the SiO_2_ surfaces (**Fig. S21b**).


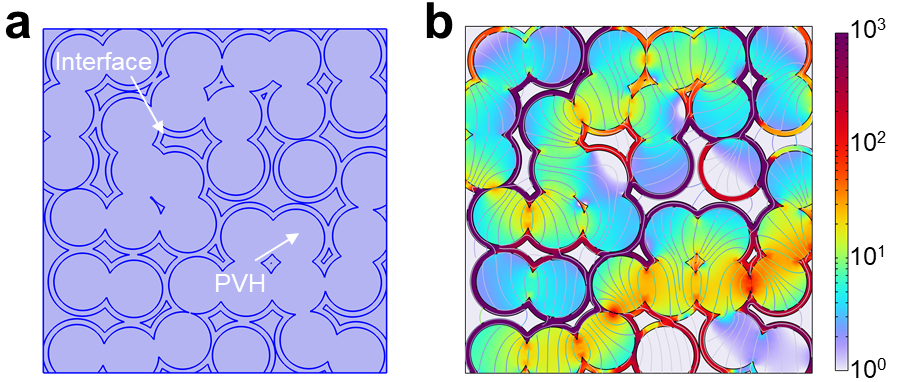


**Fig. S22** (**a**) PVH-in-SiO_2_ model and (**b**) Distribution of current densities in PVH-in-SiO_2_ model (The unit of current density is mA cm^−2^)


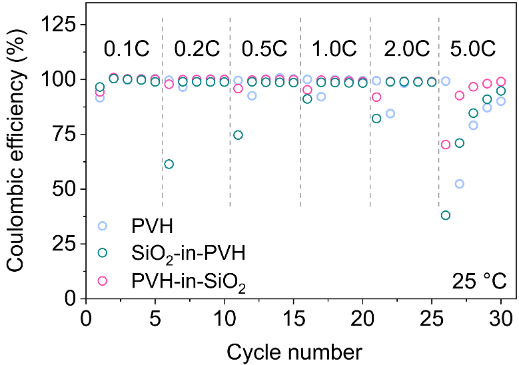


**Fig. S23** Coulombic efficiency of LFP|PVH|Li, LFP|SiO_2_-in-PVH|Li, and LFP|PVH-in-SiO_2_|Li full cells at various current densities under 25 °C


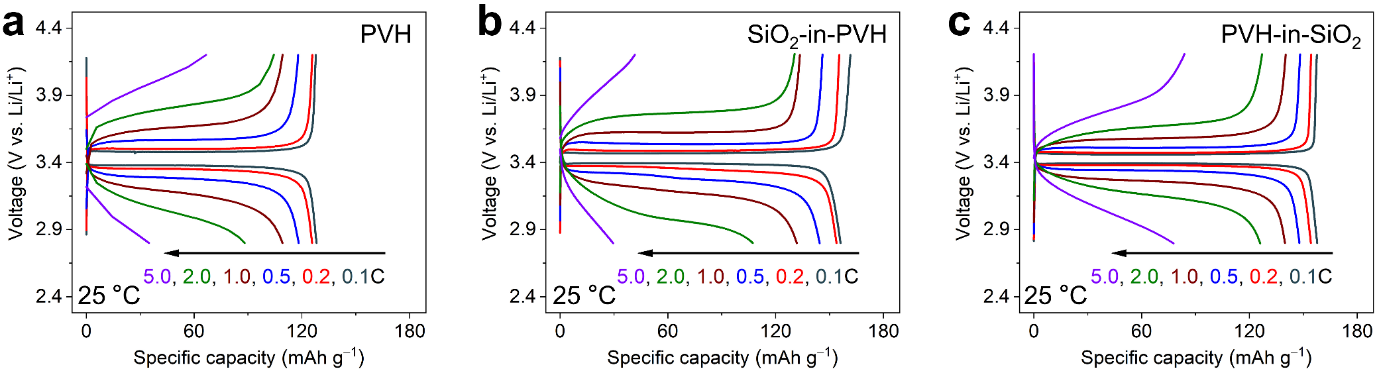


**Fig. S24** GCD curves of (**a**) LFP|PVH|Li, (**b**) LFP|SiO_2_-in-PVH|Li, and (**c**) LFP|PVH-in-SiO_2_|Li full cells at various current densities under 25 °C


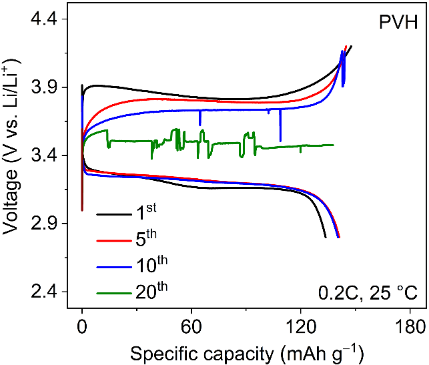


**Fig. S25** GCD curves of LFP|PVH|Li full cells at 0.2C under 25 °C


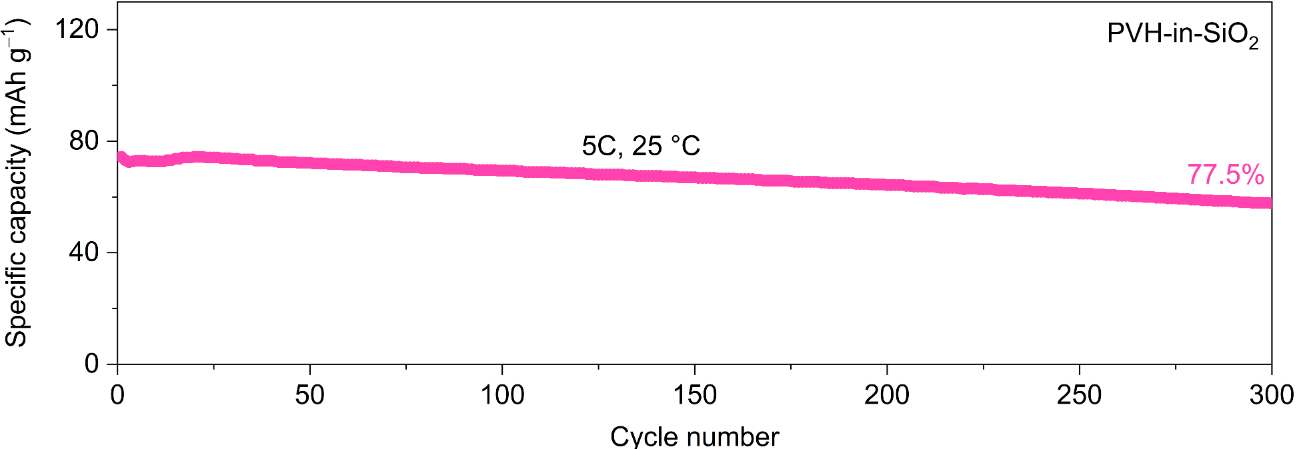


**Fig. S26** Cycling performance of LFP|PVH-in-SiO_2_|Li full cells at the current density of 5C under 25 °C

**Table S4** Comparison of specific capacity retention of our PVH-in-SiO_2_-based full cells with recently reported solid-state full cells using polymer-based solid electrolytes

| Solid-state electrolytes | Rate(C) | Cycle number | Capacity retention (%) | Refs. |
| --- | --- | --- | --- | --- |
| PVH-in-SiO_2_ | **3** | **300** | **92.9** | **This work** |
| BTO/LLTO/PVDF | 0.5 | 200 | 91.3 | [S44] |
| Defective PVH | 1 | 300 | 94.9 | [S45] |
| 2D SiO_2_/PVH | 1 | 1000 | 72.9 | [S46] |
| LATP/SN/PEO/PVH | 0.5 | 100 | 96.8 | [S61] |
| H-ZIF-8/HN | 0.1 | 200 | 84 | [S62] |
| PCIL-LZSP/PVDF | 0.5 | 1000 | 88.5 | [S43] |
| BC-g-PLiSTFSI-b-PEGM/P | 1 | 300 | 83.7 | [S63] |
| PTF-4EO | 0.5 | 200 | 89.9 | [S64] |
| GO-g-PSSLi | 1 | 170 | 86 | [S65] |
| LLZO/EmimFSI/PMMA | 1 | 300 | 90.4 | [S66] |
| Poly(VEC10-r-LiSTFSI) | 0.5 | 250 | 95 | [S67] |
| PEGDA/PVH | 0.5 | 300 | 97 | [S68] |
| LiBTFSI/PEO | 0.33 | 200 | 86.1 | [S69] |
| PAEV | 1 | 300 | 80.6 | [S70] |
| AO-PIM-1-Li | 0.2 | 200 | 93.9 | [S71] |
| Fe_3_O_4_@mSiO_2_/PVDF/PEGDA | 0.5 | 700 | 89.3 | [S72] |
| Li^+^@PI-TMEFB-COFs | 0.5 | 400 | 82 | [S73] |
| Li^+^-implanted CuMH | 0.5 | 573 | 80 | [S74] |


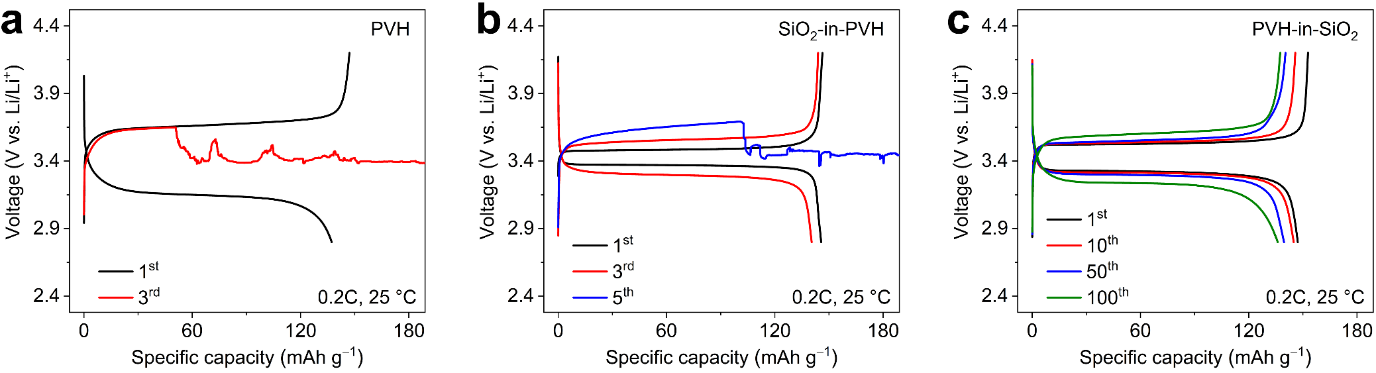


**Fig. S27** GCD curves of (**a**) LFP|PVH|Li, (**b**) LFP|SiO_2_-in-PVH|Li, and (**c**) LFP|PVH-in-SiO_2_|Li full cells at high mass loadings under 25 °C (current density: 0.2C)


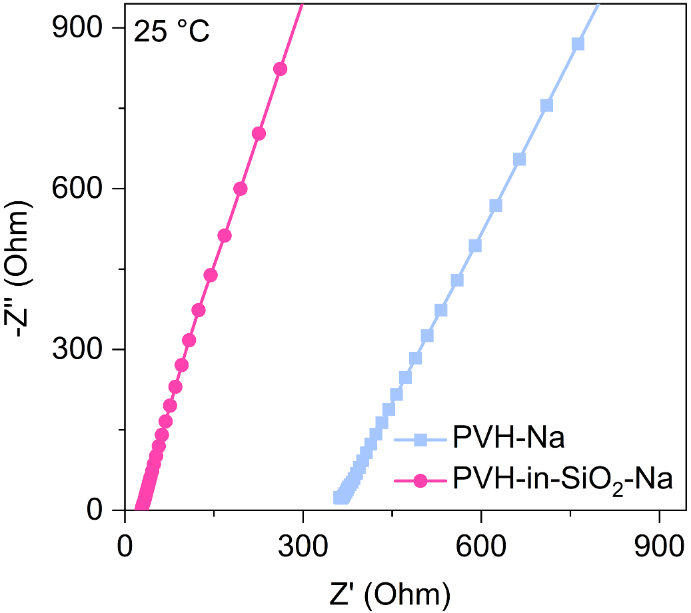


**Fig. S28** EIS curves of SS|CSEs|SS symmetric cells using PVH-Na and PVH-in-SiO_2_-Na as CSEs at 25 °C. The corresponding ionic conductivities were calculated and plotted in **Fig. 5b**


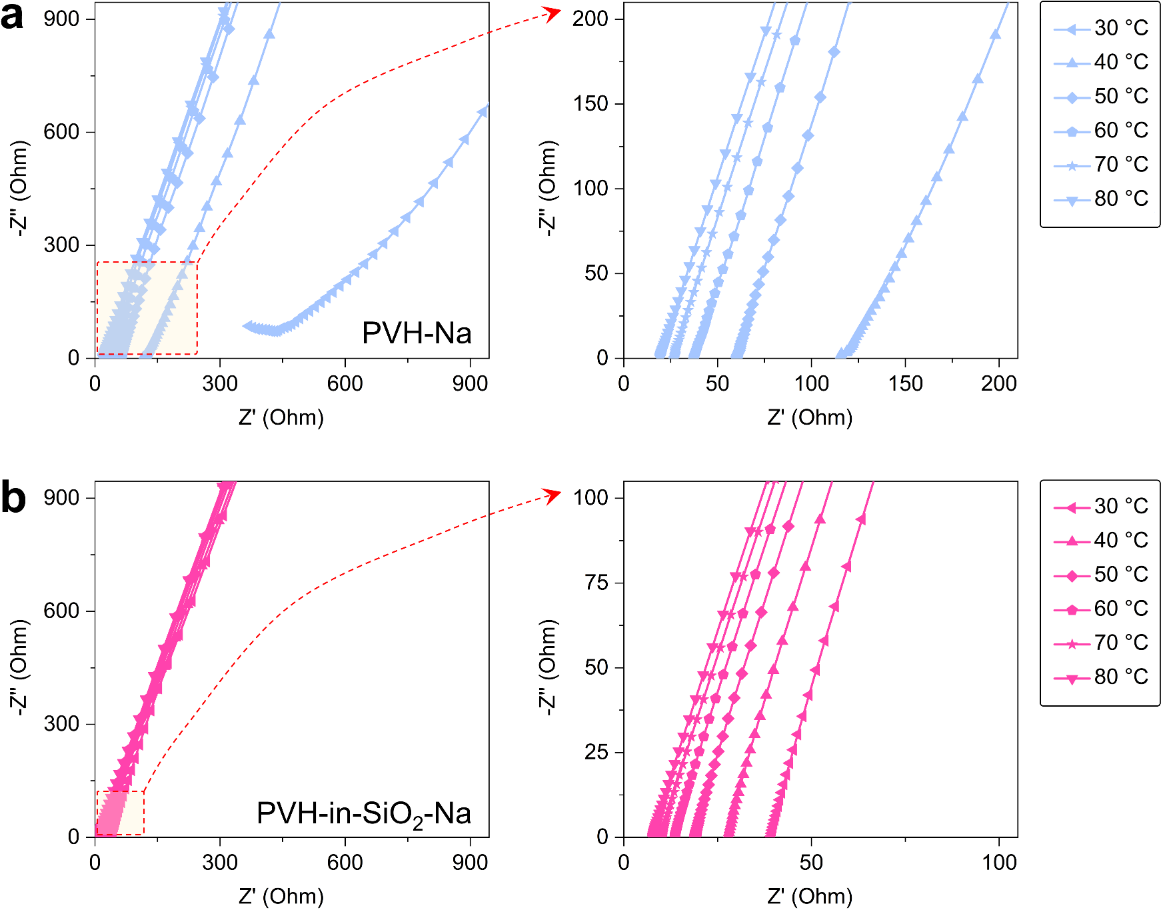


**Fig. S29** EIS curves of SS|CSEs|SS symmetric cells using (**a**) PVH-Na and (**b**) PVH-in-SiO_2_-Na as CSEs at different temperatures. The corresponding ionic conductivities and activation energies were calculated and plotted in the inset of **Fig. 5b**


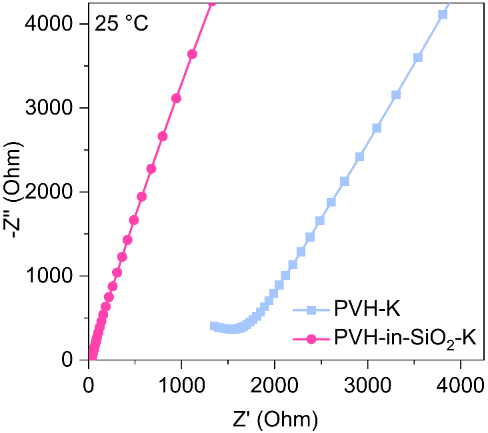


**Fig. S30** EIS curves of SS|CSEs|SS symmetric cells using PVH-K and PVH-in-SiO_2_-K as CSEs at 25 °C. The corresponding ionic conductivities were calculated and plotted in **Fig. 5d**


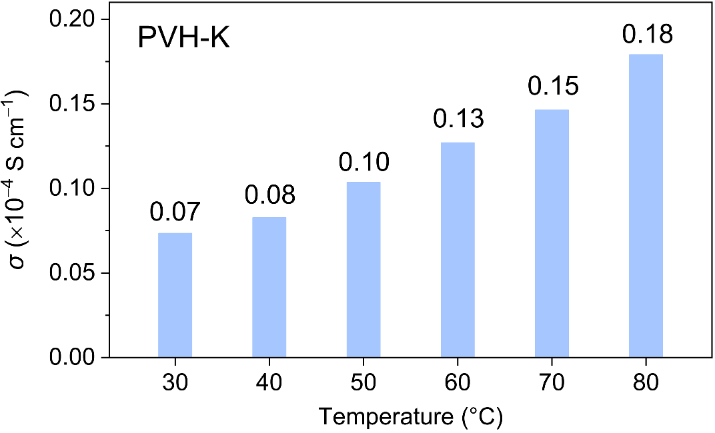


**Fig. S31** Ionic conductivity of PVH-K at different temperatures. Although we can calculate the ionic conductivities according to the corresponding EIS curves in **Fig. S24**, they are not too small to be reliable owing to the abnormal EIS curves


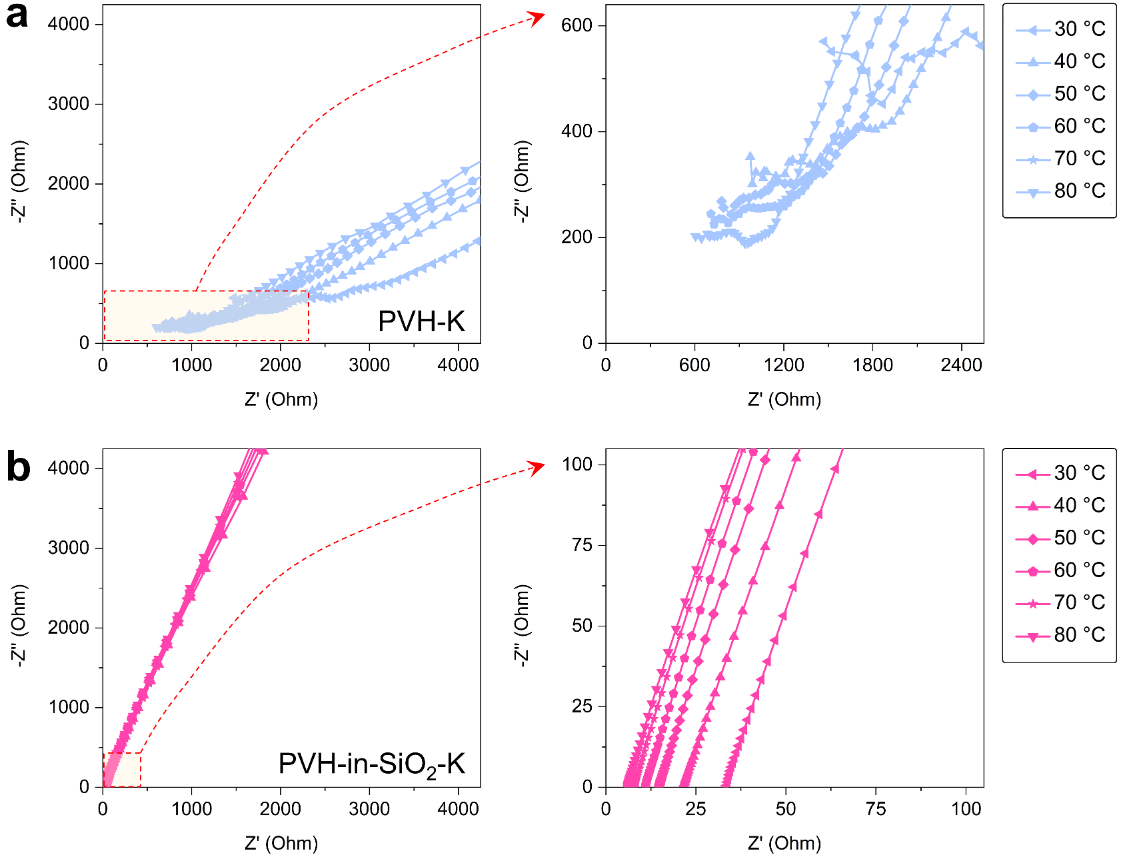


**Fig. S32** EIS curves of SS|CSEs|SS symmetric cells using (a) PVH-K and (b) PVH-in-SiO_2_-K as CSEs at different temperatures. The corresponding ionic conductivities of PVH-K and PVH-in-SiO_2_-K were calculated and plotted in the inset **Fig. 5d** and in **Fig. S23**, respectively. Note that the EIS curves of PVH-K are abnormal because of its extremely poor ionic conducting properties. Therefore, it is not scientifically necessary to calculate the activation energy of PVH-K


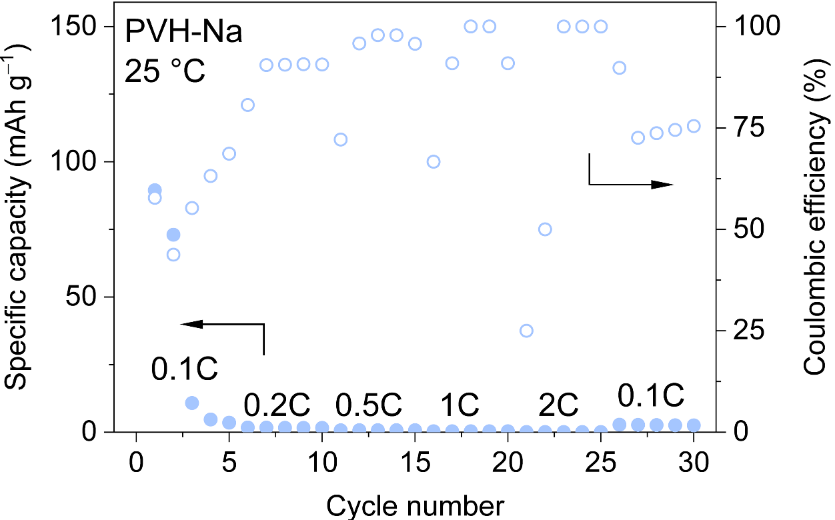


**Fig. S33** Rate capability at various current densities of NVP|PVH-Na|Na full cells at 0.5C


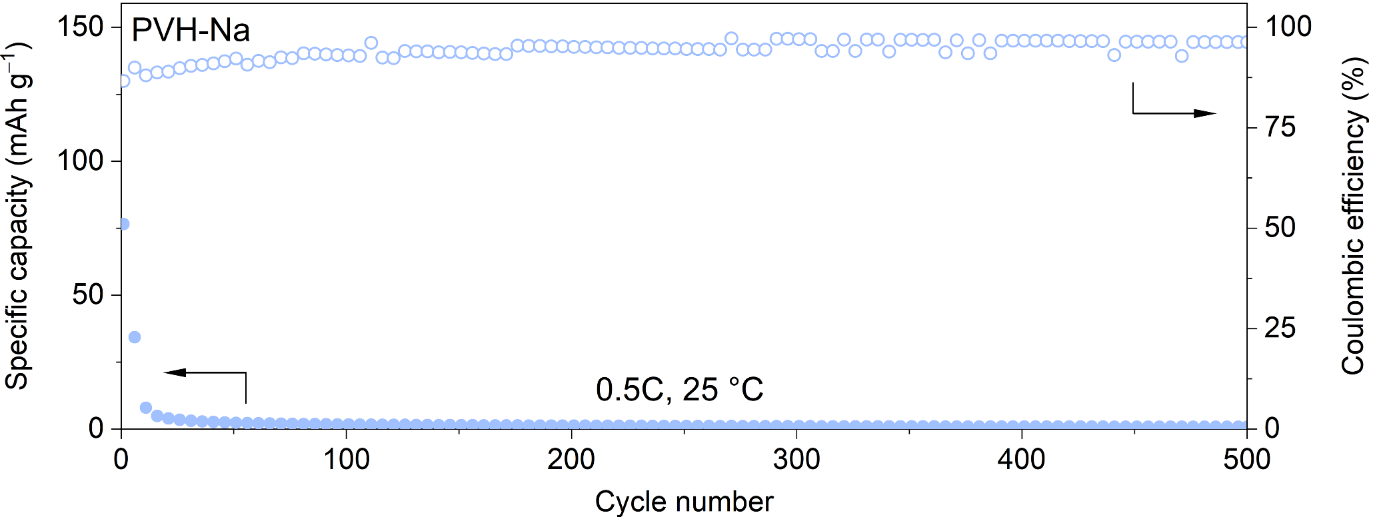


**Fig. S34** Cycling stability of NVP|PVH-Na|Na full cells at 0.5C

**Supplementary References**

1. W. Stöber, A. Fink, E. Bohn. Controlled growth of monodisperse silica spheres in the micron size range. J. Colloid Interface Sci. **26**, 62-69 (1968). <https://doi.org/10.1016/0021-9797(68)90272-5>
2. G. Kresse, J. Furthmüller. Efficient iterative schemes for ab initio total-energy calculations using a plane-wave basis set. Phys. Rev. B **54**, 11169-11186 (1996). <https://doi.org/10.1103/PhysRevB.54.11169>
3. J. P. Perdew, K. Burke, M. Ernzerhof. Generalized gradient approximation made simple. Phys. Rev. Lett. **77**, 3865-3868 (1996). <https://doi.org/10.1103/PhysRevLett.77.3865>
4. G. Kresse, D. Joubert. From ultrasoft pseudopotentials to the projector augmented-wave method. Phys. Rev. B **59**, 1758-1775 (1999). <https://doi.org/10.1103/PhysRevB.59.1758>
5. P. E. Blöchl. Projector augmented-wave method. Phys. Rev. B **50**, 17953-17979 (1994). <https://doi.org/10.1103/PhysRevB.50.17953>
6. S. Grimme, J. Antony, S. Ehrlich, H. Krieg. A consistent and accurate ab initio parametrization of density functional dispersion correction (dft-d) for the 94 elements H-Pu. J. Chem. Phys. **132**, 154104 (2010). <https://doi.org/10.1063/1.3382344>
7. M. J. Abraham, T. Murtola, R. Schulz, S. Páll, J. C. Smith et al., Gromacs: High performance molecular simulations through multi-level parallelism from laptops to supercomputers. SoftwareX **1-2**, 19-25 (2015). <https://doi.org/10.1016/j.softx.2015.06.001>
8. J. Wang, R. M. Wolf, J. W. Caldwell, P. A. Kollman, D. A. Case. Development and testing of a general amber force field. J. Comput. Chem. **25**, 1157-1174 (2004). <https://doi.org/https://doi.org/10.1002/jcc.20035>
9. T. Lu, F. Chen. Multiwfn: A multifunctional wavefunction analyzer. J. Comput. Chem. **33**, 580-592 (2012). <https://doi.org/https://doi.org/10.1002/jcc.22885>
10. R. Semino, G. Zaldívar, E. J. Calvo, D. Laria. Lithium solvation in dimethyl sulfoxide-acetonitrile mixtures. J. Chem. Phys. **141**, (2014). <https://doi.org/10.1063/1.4902837>
11. F. S. Emami, V. Puddu, R. J. Berry, V. Varshney, S. V. Patwardhan et al., Force field and a surface model database for silica to simulate interfacial properties in atomic resolution. Chem. Mater. **26**, 2647-2658 (2014). <https://doi.org/10.1021/cm500365c>
12. K. V. Kravchyk, D. T. Karabay, M. V. Kovalenko. On the feasibility of all-solid-state batteries with LLZO as a single electrolyte. Sci. Rep. **12**, 1177 (2022). <https://doi.org/10.1038/s41598-022-05141-x>
13. A. Sharafi, C. G. Haslam, R. D. Kerns, J. Wolfenstine, J. Sakamoto. Controlling and correlating the effect of grain size with the mechanical and electrochemical properties of Li_7_La_3_Zr_2_O_12_ solid-state electrolyte. J. Mater. Chem. A **5**, 21491-21504 (2017). <https://doi.org/10.1039/C7TA06790A>
14. Y.-H. Cho, J. Wolfenstine, E. Rangasamy, H. Kim, H. Choe et al., Mechanical properties of the solid Li-ion conducting electrolyte: Li_0.33_La_0.57_TiO_3_. J. Mater. Sci. **47**, 5970-5977 (2012). <https://doi.org/10.1007/s10853-012-6500-5>
15. K.-Y. Yang, J.-W. Wang, K.-Z. Fung. Roles of lithium ions and La/Li-site vacancies in sinterability and total ionic conduction properties of polycrystalline Li_3x_La_2/3−x_TiO_3_ solid electrolytes (0.21≤3x≤0.50). J. Alloys. Compd. **458**, 415-424 (2008). <https://doi.org/10.1016/j.jallcom.2007.03.130>
16. J. S. Thokchom, B. Kumar. Composite effect in superionically conducting lithium aluminium germanium phosphate based glass-ceramic. J. Power Sources **185**, 480-485 (2008). <https://doi.org/10.1016/j.jpowsour.2008.07.009>
17. Y. Zhu, T. Wu, J. Sun, M. Kotobuki. Highly conductive lithium aluminum germanium phosphate solid electrolyte prepared by sol-gel method and hot-pressing. Solid State Ionics **350**, 115320 (2020). <https://doi.org/https://doi.org/10.1016/j.ssi.2020.115320>
18. H. Chung, B. Kang. Increase in grain boundary ionic conductivity of Li_1.5_Al_0.5_Ge_1.5_(PO_4_)_3_ by adding excess lithium. Solid State Ionics **263**, 125-130 (2014). <https://doi.org/10.1016/j.ssi.2014.05.016>
19. H. Zhu, A. Prasad, S. Doja, L. Bichler, J. Liu. Spark plasma sintering of lithium aluminum germanium phosphate solid electrolyte and its electrochemical properties. Nanomaterials **9**, 1086 (2019). <https://doi.org/10.3390/nano9081086>
20. Y. Ren, H. Deng, H. Zhao, Z. Zhou, Z. Wei. A simple and effective method to prepare dense Li_1.3_Al_0.3_Ti_1.7_(PO_4_)_3_ solid–state electrolyte for lithium-oxygen batteries. Ionics **26**, 6049-6056 (2020). <https://doi.org/10.1007/s11581-020-03781-5>
21. K. Waetzig, A. Rost, C. Heubner, M. Coeler, K. Nikolowski et al., Synthesis and sintering of Li_1.3_Al_0.3_Ti_1.7_(PO_4_)_3_ (LATP) electrolyte for ceramics with improved Li^+^ conductivity. J. Alloys. Compd. **818**, 153237 (2020). <https://doi.org/10.1016/j.jallcom.2019.153237>
22. S. Duluard, A. Paillassa, L. Puech, P. Vinatier, V. Turq et al., Lithium conducting solid electrolyte Li_1.3_Al_0.3_Ti_1.7_(PO_4_)_3_ obtained via solution chemistry. J. Eur. Ceram. Soc. **33**, 1145-1153 (2013). <https://doi.org/10.1016/j.jeurceramsoc.2012.08.005>
23. Y. Wang, B. Hoang, J. Hoerauf, C. Lee, C.-F. Lin et al., Hot and cold pressed lgps solid electrolytes. J. Electrochem. Soc. **168**, 010533 (2021). <https://doi.org/10.1149/1945-7111/abdb44>
24. S. Wang, X. Zhang, S. Liu, C. Xin, C. Xue et al., High-conductivity free-standing Li_6_PS_5_Cl/poly(vinylidene difluoride) composite solid electrolyte membranes for lithium-ion batteries. J. Materiomics **6**, 70-76 (2020). [https://doi.org/10.1016/j.jmat.2019.12.010](https://doi.org/https://doi.org/10.1016/j.jmat.2019.12.010)
25. Y. Huh, H. Gon Lee, C.-M. Cho, J.-W. Park, B. Gon Kim et al., Solution-processed synthesis of nano-sized argyrodite solid electrolytes with cavitation effect for high performance all-solid-state lithium-ion batteries. Batteries Supercaps **6**, e202300036 (2023). <https://doi.org/10.1002/batt.202300036>
26. C. M. Combes, P. Dorenbos, C. W. E. van Eijk, K. W. Krämer, H. U. Güdel. Optical and scintillation properties of pure and Ce^3+^-doped Cs_2_LiYCl_6_ and Li_3_YCl_6_:Ce^3+^ crystals. J. Lumin. **82**, 299-305 (1999). <https://doi.org/10.1016/S0022-2313(99)00047-2>
27. C. Murray, C. Flannery, I. Streiter, S. E. Schulz, M. R. Baklanov et al., Comparison of techniques to characterise the density, porosity and elastic modulus of porous low-k SiO_2_ xerogel films. Microelectron. Eng. **60**, 133-141 (2002). <https://doi.org/10.1016/S0167-9317(01)00589-5>
28. R. R. Madathingal, S. L. Wunder. Thermal degradation of PEO on SiO_2_ nanoparticles as a function of SiO_2_ silanol density, hydrophobicity and size. Thermochim. Acta **523**, 182-186 (2011). <https://doi.org/10.1016/j.tca.2011.05.022>
29. H. Hashimoto, Y. Onodera, S. Tahara, S. Kohara, K. Yazawa et al., Structure of alumina glass. Sci. Rep. **12**, 516 (2022). <https://doi.org/10.1038/s41598-021-04455-6>
30. T. K. Phung, C. Herrera, M. Á. Larrubia, M. García-Diéguez, E. Finocchio et al., Surface and catalytic properties of some γ-Al_2_O_3_ powders. Appl. Catal. A **483**, 41-51 (2014). <https://doi.org/10.1016/j.apcata.2014.06.020>
31. S. Kim, R. Hidayat, H. Roh, J. Kim, H.-L. Kim et al., Atomic layer deposition of titanium oxide thin films using a titanium precursor with a linked amido-cyclopentadienyl ligand. J. Mater. Chem. C. **10**, 6696-6709 (2022). <https://doi.org/10.1039/D2TC00574C>
32. A. Jolivet, C. Labbé, C. Frilay, O. Debieu, P. Marie et al., Structural, optical, and electrical properties of TiO_2_ thin films deposited by ald: Impact of the substrate, the deposited thickness and the deposition temperature. Appl. Surf. Sci. **608**, 155214 (2023). <https://doi.org/10.1016/j.apsusc.2022.155214>
33. C. Zhang, Z. Feng, Y. Zhang, Z. Xia, N. Hakimi et al., Structural evolution of mgo layer in Mg-based composites reinforced by metallic glasses during the sps sintering process. Vacuum **214**, 112141 (2023). <https://doi.org/10.1016/j.vacuum.2023.112141>
34. K. L. Meena, C. S. Vidyasagar, D. Benny Karunakar. Mechanical and tribological properties of mgo/multiwalled carbon nanotube-reinforced zirconia-toughened alumina composites developed through spark plasma sintering and microwave sintering. J. Mater. Eng. Perform. **31**, 682-696 (2022). <https://doi.org/10.1007/s11665-021-06170-9>
35. D. K. Sharma, S. Shukla, K. K. Sharma, V. Kumar. A review on ZnO: Fundamental properties and applications. Mater. Today: Proc **49**, 3028-3035 (2022). <https://doi.org/10.1016/j.matpr.2020.10.238>
36. A. Galan-Gonzalez, A. Gallant, D. A. Zeze, D. Atkinson. Controlling the growth of single crystal ZnO nanowires by tuning the atomic layer deposition parameters of the ZnO seed layer. Nanotechnology **30**, 305602 (2019). <https://doi.org/10.1088/1361-6528/ab186a>
37. D. Lei, N. Hu, L. Wu, Alamusi, H. Ning et al., Improvement of the piezoelectricity of PVDF-HFP by CoFe_2_O_4_ nanoparticles. Nano Mater. Sci. 201-210 (2023). <https://doi.org/10.1016/j.nanoms.2023.03.002>
38. H. Huo, Y. Chen, J. Luo, X. Yang, X. Guo et al., Rational design of hierarchical “ceramic-in-polymer” and “polymer-in-ceramic” electrolytes for dendrite-free solid-state batteries. Adv. Energy Mater. **9**, 1804004 (2019). <https://doi.org/10.1002/aenm.201804004>
39. Z. Li, F. Liu, S. Chen, F. Zhai, Y. Li et al., Single Li ion conducting solid-state polymer electrolytes based on carbon quantum dots for Li-metal batteries. Nano Energy **82**, 105698 (2021). <https://doi.org/10.1016/j.nanoen.2020.105698>
40. Z. Zhang, S. Zhang, S. Geng, S. Zhou, Z. Hu et al., Agglomeration-free composite solid electrolyte and enhanced cathode-electrolyte interphase kinetics for all-solid-state lithium metal batteries. Energy Storage Mater. **51**, 19-28 (2022). <https://doi.org/10.1016/j.ensm.2022.06.025>
41. Y. Jin, X. Zong, X. Zhang, Z. Jia, H. Xie et al., Constructing 3D Li^+^-percolated transport network in composite polymer electrolytes for rechargeable quasi-solid-state lithium batteries. Energy Storage Mater. **49**, 433-444 (2022). <https://doi.org/10.1016/j.ensm.2022.04.035>
42. J. Yu, G. Zhou, Y. Li, Y. Wang, D. Chen, F. Ciucci. Improving room-temperature Li-metal battery performance by in situ creation of fast Li^+^ transport pathways in a polymer-ceramic electrolyte. Small **19**, 2302691 (2023). <https://doi.org/10.1002/smll.202302691>
43. L. Zhu, J. Chen, Y. Wang, W. Feng, Y. Zhu et al., Tunneling interpenetrative lithium ion conduction channels in polymer-in-ceramic composite solid electrolytes. J. Am. Chem. Soc. **146**, 6591-6603 (2024). <https://doi.org/10.1021/jacs.3c11988>
44. P. Shi, J. Ma, M. Liu, S. Guo, Y. Huang et al., A dielectric electrolyte composite with high lithium-ion conductivity for high-voltage solid-state lithium metal batteries. Nat. Nanotechnol. **18**, 602-610 (2023). <https://doi.org/10.1038/s41565-023-01341-2>
45. C. Dai, M. Weng, B. Cai, J. Liu, S. Guo et al., Ion-conductive crystals of poly (vinylidene fluoride) enables fast charging solid-state lithium metal batteries. Energy Environ. Sci. (2024). <https://doi.org/10.1039/D4EE03467H>
46. Z. Luo, W. Li, C. Guo, Y. Song, M. Zhou et al., Two-dimensional silica enhanced solid polymer electrolyte for lithium metal batteries. Particuology **85**, 146-154 (2024). <https://doi.org/10.1016/j.partic.2023.04.002>
47. X. An, Y. Liu, K. Yang, J. Mi, J. Ma et al., Dielectric filler-induced hybrid interphase enabling robust solid-state Li metal batteries at high areal capacity. Adv. Mater. **36**, 2311195 (2024). <https://doi.org/10.1002/adma.202311195>
48. S. Lv, X. He, Z. Ji, S. Yang, L. Feng et al., A supertough and highly-conductive nano-dipole doped composite polymer electrolyte with hybrid Li^+^-solvation microenvironment for lithium metal batteries. Adv. Energy Mater. **13**, 2302711 (2023). <https://doi.org/10.1002/aenm.202302711>
49. H. Cheng, D. Li, B. Xu, Y. Wei, H. Wang et al., Amorphous silicon nitride induced high dielectric constant toward long-life solid lithium metal battery. Energy Storage Mater. **53**, 305-314 (2022). <https://doi.org/10.1016/j.ensm.2022.09.003>
50. B.-H. Kang, S.-F. Li, J. Yang, Z.-M. Li, Y.-F. Huang. Uniform lithium plating for dendrite-free lithium metal batteries: Role of dipolar channels in poly(vinylidene fluoride) and PbZr_x_Ti_1–x_O_3_ interface. ACS Nano **17**, 14114-14122 (2023). <https://doi.org/10.1021/acsnano.3c04684>
51. K. Yang, L. Chen, J. Ma, C. Lai, Y. Huang et al., Stable interface chemistry and multiple ion transport of composite electrolyte contribute to ultra-long cycling solid-state LiNi_0.8_Co_0.1_Mn_0.1_O_2_/lithium metal batteries. Angew. Chem., Int. Ed. **60**, 24668-24675 (2021). <https://doi.org/10.1002/anie.202110917>
52. X. Zhang, S. Wang, C. Xue, C. Xin, Y. Lin et al., Self-suppression of lithium dendrite in all-solid-state lithium metal batteries with poly(vinylidene difluoride)-based solid electrolytes. Adv. Mater. **31**, 1806082 (2019). <https://doi.org/10.1002/adma.201806082>
53. W. Liu, C. Yi, L. Li, S. Liu, Q. Gui et al., Designing polymer-in-salt electrolyte and fully infiltrated 3D electrode for integrated solid-state lithium batteries. Angew. Chem., Int. Ed. **60**, 12931-12940 (2021). <https://doi.org/10.1002/anie.202101537>
54. H. Sun, X. Xie, Q. Huang, Z. Wang, K. Chen et al., Fluorinated poly-oxalate electrolytes stabilizing both anode and cathode interfaces for all-solid-state Li/NMC811 batteries. Angew. Chem., Int. Ed. **60**, 18335-18343 (2021). <https://doi.org/10.1002/anie.202107667>
55. J. Mi, J. Ma, L. Chen, C. Lai, K. Yang et al., Topology crafting of polyvinylidene difluoride electrolyte creates ultra-long cycling high-voltage lithium metal solid-state batteries. Energy Storage Mater. **48**, 375-383 (2022). <https://doi.org/10.1016/j.ensm.2022.02.048>
56. Y.-F. Huang, T. Gu, G. Rui, P. Shi, W. Fu et al., A relaxor ferroelectric polymer with an ultrahigh dielectric constant largely promotes the dissociation of lithium salts to achieve high ionic conductivity. Energy Environ. Sci. **14**, 6021-6029 (2021). <https://doi.org/10.1039/D1EE02663A>
57. L. Wu, F. Pei, D. Cheng, Y. Zhang, H. Cheng et al., Flame-retardant polyurethane-based solid-state polymer electrolytes enabled by covalent bonding for lithium metal batteries. Adv. Func. Mater. **34**, 2310084 (2024). <https://doi.org/10.1002/adfm.202310084>
58. T. Deng, L. Cao, X. He, A.-M. Li, D. Li et al., In situ formation of polymer-inorganic solid-electrolyte interphase for stable polymeric solid-state lithium-metal batteries. Chem **7**, 3052-3068 (2021). <https://doi.org/10.1016/j.chempr.2021.06.019>
59. W. Yang, Y. Liu, X. Sun, Z. He, P. He et al., Solvation-tailored PVDF-based solid-state electrolyte for high-voltage lithium metal batteries. Angew. Chem., Int. Ed. **63**, e202401428 (2024). <https://doi.org/10.1002/anie.202401428>
60. H. Wang, H. Cheng, D. Li, F. Li, Y. Wei et al., Lithiated copper polyphthalocyanine with extended π-conjugation induces LiF-rich solid electrolyte interphase toward long-life solid-state lithium-metal batteries. Adv. Energy Mater. **13**, 2204425 (2023). <https://doi.org/10.1002/aenm.202204425>
61. Y. Hu, L. Li, H. Tu, X. Yi, J. Wang et al., Janus electrolyte with modified Li^+^ solvation for high-performance solid-state lithium batteries. Adv. Func. Mater. **32**, 2203336 (2022). <https://doi.org/10.1002/adfm.202203336>
62. F. Tao, X. Wang, S. Jin, L. Tian, Z. Liu et al., A composite of hierarchical porous mofs and halloysite nanotubes as single-ion-conducting electrolyte toward high-performance solid-state lithium-ion batteries. Adv. Mater. **35**, 2300687 (2023). <https://doi.org/10.1002/adma.202300687>
63. M. Zhou, R. Liu, D. Jia, Y. Cui, Q. Liu et al., Ultrathin yet robust single lithium-ion conducting quasi-solid-state polymer-brush electrolytes enable ultralong-life and dendrite-free lithium-metal batteries. Adv. Mater. **33**, 2100943 (2021). <https://doi.org/10.1002/adma.202100943>
64. H. Li, Y. Du, Q. Zhang, Y. Zhao, F. Lian. A single-ion conducting network as rationally coordinating polymer electrolyte for solid-state Li metal batteries. Adv. Energy Mater. **12**, 2103530 (2022). <https://doi.org/10.1002/aenm.202103530>
65. Q. Liu, R. Liu, Y. Cui, M. Zhou, J. Zeng et al., Dendrite-free and long-cycling lithium metal battery enabled by ultrathin, 2D shield-defensive, and single lithium-ion conducting polymeric membrane. Adv. Mater. **34**, 2108437 (2022). <https://doi.org/10.1002/adma.202108437>
66. Y. Zhai, W. Hou, M. Tao, Z. Wang, Z. Chen et al., Enabling high-voltage “superconcentrated ionogel-in-ceramic” hybrid electrolyte with ultrahigh ionic conductivity and single Li^+^-ion transference number. Adv. Mater. **34**, 2205560 (2022). <https://doi.org/10.1002/adma.202205560>
67. X. Shan, M. Morey, Z. Li, S. Zhao, S. Song et al., A polymer electrolyte with high cationic transport number for safe and stable solid Li-metal batteries. ACS Energy Lett. **7**, 4342-4351 (2022). <https://doi.org/10.1021/acsenergylett.2c02349>
68. X. Zuo, Y. Cheng, L. Xu, R. Chen, F. Liu et al., A novel thioctic acid-functionalized hybrid network for solid-state batteries. Energy Storage Mater. **46**, 570-576 (2022). <https://doi.org/10.1016/j.ensm.2022.01.045>
69. L. Qiao, S. Rodriguez Peña, M. Martínez-Ibañez, A. Santiago, I. Aldalur et al., Anion π–π stacking for improved lithium transport in polymer electrolytes. J. Am. Chem. Soc. **144**, 9806-9816 (2022). <https://doi.org/10.1021/jacs.2c02260>
70. Z. Tian, L. Hou, D. Feng, Y. Jiao, P. Wu. Modulating the coordination environment of lithium bonds for high performance polymer electrolyte batteries. ACS Nano **17**, 3786-3796 (2023). <https://doi.org/10.1021/acsnano.2c11734>
71. X.-X. Wang, L.-N. Song, L.-J. Zheng, D.-H. Guan, C.-L. Miao et al., Polymers with intrinsic microporosity as solid ion conductors for solid-state lithium batteries. Angew. Chem., Int. Ed. **62**, e202308837 (2023). <https://doi.org/10.1002/anie.202308837>
72. T. Deng, Q. Han, J. Liu, C. Yang, J. Wang et al., Vertically aligned hollow mesoporous silica rods enabling composite polymer electrolytes with fast ionic conduction for lithium metal batteries. Adv. Func. Mater. **34**, 2311952 (2024). <https://doi.org/10.1002/adfm.202311952>
73. Y. Yuan, Z. Zhang, Z. Zhang, K.-T. Bang, Y. Tian et al., Highly conductive imidazolate covalent organic frameworks with ether chains as solid electrolytes for lithium metal batteries. Angew. Chem, Int. Ed. **63**, e202402202 (2024). <https://doi.org/10.1002/anie.202402202>
74. X. Zhan, M. Li, X. Zhao, Y. Wang, S. Li et al., Self-assembled hydrated copper coordination compounds as ionic conductors for room temperature solid-state batteries. Nat. Commun. **15**, 1056 (2024). <https://doi.org/10.1038/s41467-024-45372-2>
